# Multidisciplinary methodologies used in the study of cable bacteria

**DOI:** 10.1093/femsre/fuae030

**Published:** 2024-12-13

**Authors:** Michaela M H Wawryk, Philip Ley, Diana Vasquez-Cardenas, Rico F Tabor, Perran L M Cook

**Affiliations:** School of Chemistry, Monash University, Clayton 3800 VIC, Australia; Department of Biology, University of Antwerp, Wilrijk 2020, Belgium; Department of Biology, University of Antwerp, Wilrijk 2020, Belgium; School of Chemistry, Monash University, Clayton 3800 VIC, Australia; School of Chemistry, Monash University, Clayton 3800 VIC, Australia

**Keywords:** Cable bacteria, microbial community, genomics, microscopy, microprofiling, enrichment cultures

## Abstract

Cable bacteria are a unique type of filamentous microorganism that can grow up to centimetres long and are capable of long-distance electron transport over their entire lengths. Due to their unique metabolism and conductive capacities, the study of cable bacteria has required technical innovations, both in adapting existing techniques and developing entirely new ones. This review discusses the existing methods used to study eight distinct aspects of cable bacteria research, including the challenges of culturing them in laboratory conditions, performing physical and biochemical extractions, and analysing the conductive mechanism. As cable bacteria research requires an interdisciplinary approach, methods from a range of fields are discussed, such as biogeochemistry, genomics, materials science, and electrochemistry. A critical analysis of the current state of each approach is presented, highlighting the advantages and drawbacks of both commonly used and emerging methods.

## Teaser

This review discusses the range of methods used in studying cable bacteria to highlight key techniques in the field and explore areas for potential innovation.

## Introduction

**Figure 1.**
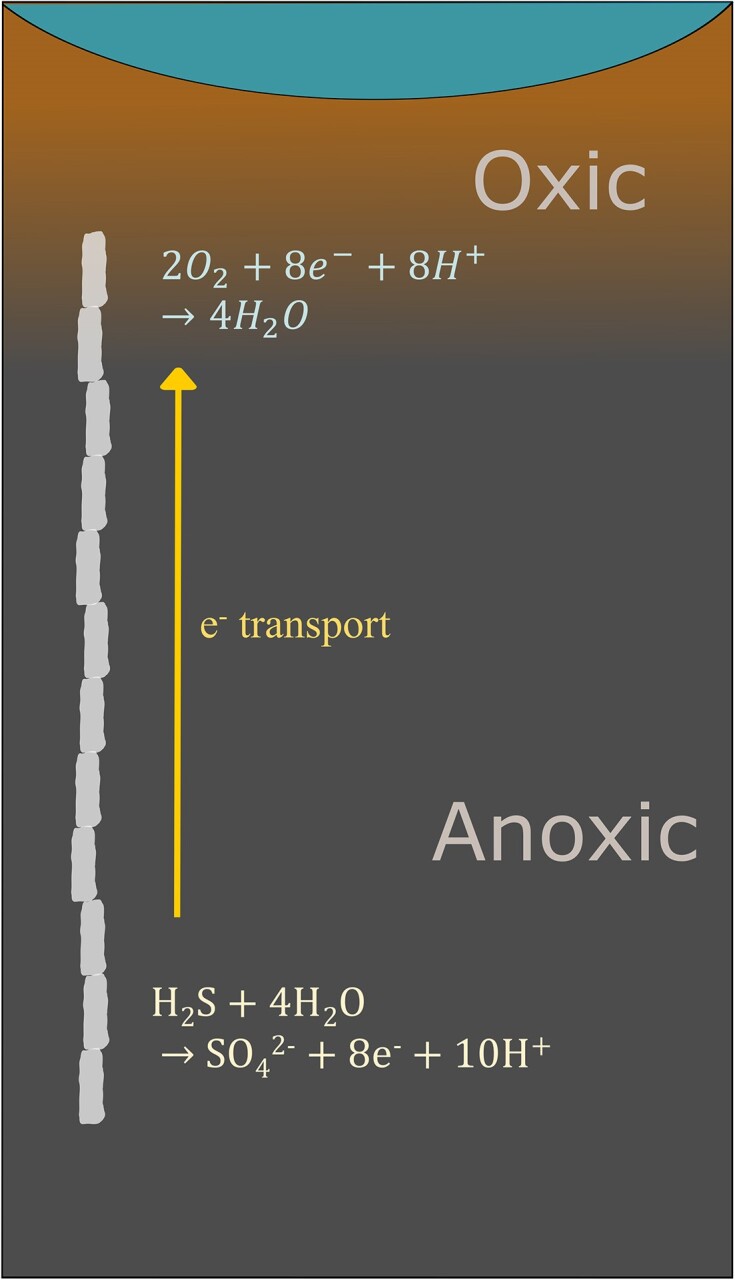
Schematic of the position of CB in sediment and the half-redox reactions they perform in the oxic and anoxic regions of sediment.

Cable bacteria (CB) are centimetre-long, multicellular bacteria found in sediment from both freshwater and marine environments all over the world (Burdorf et al. 2017, Dam et al. 2021, Geelhoed et al. 2023). They span the oxic and anoxic zones of these aquatic environments, and by conducting electrons along their filaments, CB metabolically reduce oxygen near the surface and oxidize sulfide at depth. This unique metabolism is known as electrogenic sulfide oxidation (e-SOx) (Nielsen et al. 2010, Pfeffer et al. 2012). CB utilize highly conductive, continuous fibres that run lengthwise along each filament to achieve long-distance electron transport (LDET), allowing them to complete the redox reactions (Pfeffer et al. 2012, Meysman et al. 2019) (Fig. 1). These fibres lie beneath their cell membrane, and are comprised largely of protein with trace amounts of metals such as iron, copper, and nickel (Thiruvallur Eachambadi et al. 2020, 2021, Boschker et al. 2021).

Environmentally, e-SOx has a wide range of geochemical effects within sediment (Nielsen et al. 2010, 2015, Meysman et al. 2015). Most notably is the formation of a suboxic zone in the sediment column, a region where both oxygen and sulfide are depleted due to rapid consumption by CB (Nielsen et al. 2010, Pfeffer et al. 2012). CB also acidify deeper sediment, which results in the dissolution of iron sulfide that is then oxidized near the surface, increasing iron oxides in the top few millimetres (Risgaard-Petersen et al. 2012, Meysman et al. 2015, Rao et al. 2016, Hermans et al. 2020). This enrichment of iron oxide acts as a firewall that can prevent euxinia (Seitaj et al. 2015, Sulu-Gambari et al. 2016), as well as limit phosphorous concentrations (Sulu-Gambari et al. 2016, Burdorf et al. 2024) and increase dissimilatory nitrate reduction to ammonium (DNRA) (Kessler et al. 2018). Given the complex array of effects CB can have on their environment, several studies have investigated their use in environmental applications (Dong et al. 2024). For example, sediment with active CB has been used for environmental remediation (Marzocchi et al. 2020, Xiong et al. 2024), reducing greenhouse gas emissions (Scholz et al. 2020) and electricity production within microbial fuel cells (Reimers et al. 2017); however, upscaling remains a challenge (Algar et al. 2020).

**Figure 2.**
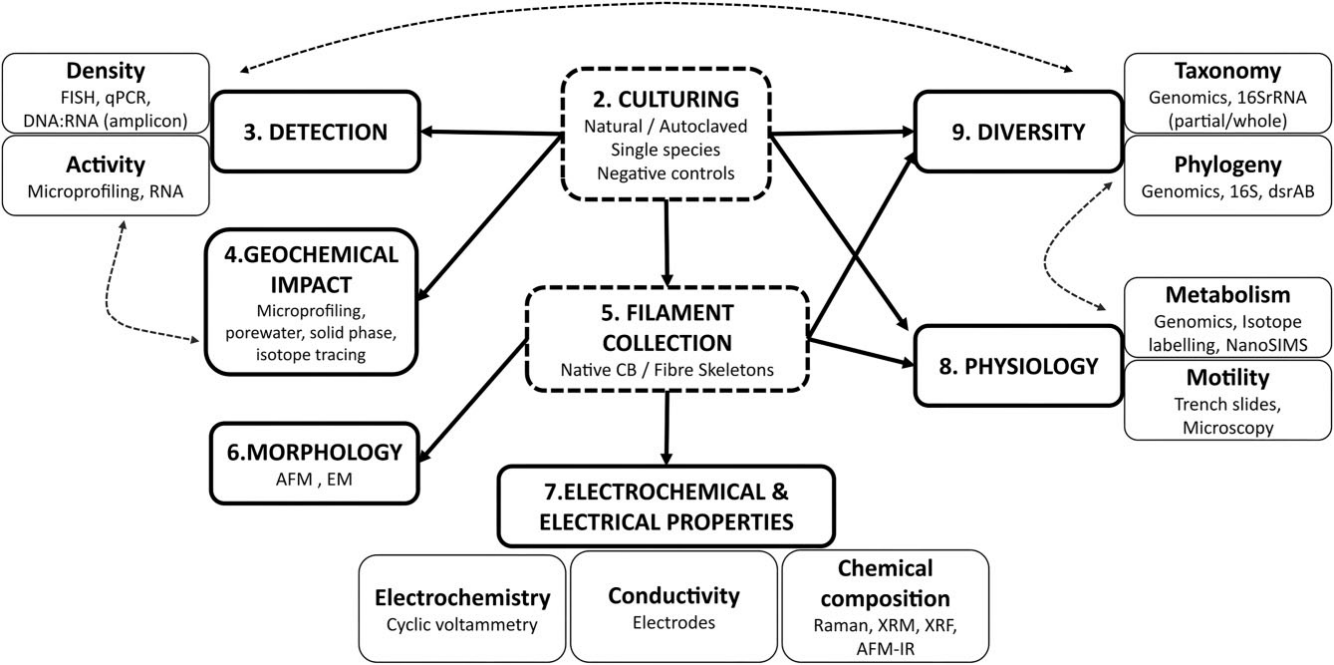
Diagram highlighting the eight major categories of CB research and the corresponding key methods discussed in this review. Culturing and filament collection are the starting points for most CB experimental designs. Potential workflows are depicted with arrows, and dashed lines link methods that are used for different categories. Advantages and disadvantages for each method are summarized in Table 1.

Since the identification of CB in 2012 (Pfeffer et al. 2012), a combination of innovation and adaptation of existing methods has allowed for progress in understanding these unique bacteria that have fundamentally shifted our knowledge of biological conductivity. This review aims to summarize and analyse the current, commonly used techniques as well as emerging methods applied in CB research, as outlined in Fig. 2. In doing so, the advantages and drawbacks of different approaches are compared (Table 1), showing the current position of CB research and highlighting areas for future development.

**Table 1.** Summary of the methods used for CB research, organized by category as outlined in the text. For each method, key conditions, advantages, and disadvantages are outlined.

Category	Method	Conditions	Advantages	Disadvantages
Culturing	Enrichment in natural sediment (Fig. 3)		Easy to do with limited equipment Diverse microbial community >1 species of CB present Viable up to 4 months	Diverse microbial community Diverse CB population Salinity must be maintained
	Enrichment culture in autoclaved sediment	Requires a source of CB from culture or field	Reduced microbial variability >1 species of CB present Good replication between cores	Alters initial sediment composition May not mimic natural conditions
	Single-strain culture	Requires a source of CB from culture or field	One CB species Simplified microbial community	Needs frequent and continuous transfers and maintenance
	Negative control—wire cutting		Immediate disruption of LDET Easy to perform Useful for short term studies Does not alter sediment conditions	Only works on short time frames (<2 days) Variable depth of cut between experiments
	Negative control—filter at 1 cm		Impedes CB growth from the start Allows for diffusion of solutes Long time frame (>20 days)	Need pore size <2 µm, which may block particles CB still present in sediment at depth CB can migrate around filter edges if not carefully placed
	Negative control—autoclaving		Simplified microbial community Eliminates active CB Allows for long-term experiments	Alters initial sediment biogeochemistry May not mimic natural conditions Largely removes microbiome
Detection	pH, sulfide, oxygen microprofiling	Electrodes require calibration	Utilizes established techniques/equipment Minimal sediment disturbance Useful to determine filament length Rapid assessment of sediment Also serves to measure geochemical impact of CB	Difficult to quantify activity accurately Microelectrodes prone to breaking in sediment May not be effective with modified sediments, e.g. high/low sulfide
	Electrical potential microprofiling	No calibration required	Minimal sediment disturbance Can provide quantitative measurement of CB activity Rapid assessment of sediment	Highly sensitive to salinity changes Microelectrodes prone to breaking in sediment Sensitive to other electrical apparatus in lab
	Fluorescence *in situ* hybridization (Fig. 6)	Core slicing	Estimates density of CB Accurate at isolating CB Can target particular species of CB in the same sample	Destructive method Requires slicing of core High variability so may need validation with another technique Quantification is time-consuming so replication not often reported
	qPCR	Core slicing	qPCR provides absolute abundance Can be more accurate than FISH RNA relative abundance serve as proxy for activity	May not target full CB diversity if species-specific probe is not known/used Destructive method
Geochemical analyses	Microprofiling		Sensors have been developed for a wide range of environmental analytes (O_2_, CO_2_, H_2_S, forms of nitrogen, methane, etc.) Provides high resolution profiles	May be more expensive to access equipment Limited to analytes dissolved in porewater
	Core slicing (porewater and sediment analysis)	Core slicing may need to be done in inert atmosphere for oxygen sensitive species	Allows for measurement of more variables (e.g. phosphorous, iron species) More accurate in determining speciation and binding equilibriums in sediment Can be more accessible than microprofiling (e.g. acid volatile sulfide assay versus H_2_S microelectrode) Measures both soluble and solid/adsorbed analytes	Destructive methods Lower-resolution profiles compared to microprofiles
	Closed core incubations	Cores must be incubated in sealed pots to ensure gas headspace is not mixed with air	Uses common analytical equipment (gas chromatography)	Short term only as oxygen may be depleted
	Isotope tracing	Typically utilizes isotope ratio mass spectrometry Must be done with elements with > 1 stable isotope, with one being more naturally abundant	Can be done for common organic elements, including nitrogen and carbon Provides unique insight into the cycles of these elements Ideal for short term experiments (<48 h)	Challenging for certain elements such as P, which have isotopes with short half-lives Some isotopes can be expensive, so thoughtful experimental design is crucial Cryptic cycling of elements may produce misleading results
Filament extractions	Hand picking filaments	Performed under microscope	Simple to perform Allows the collection of native filaments (individuals) Allows for very clean filaments to be prepared	Handling can damage filament structures Biased towards larger/more robust species Requires repeated washing to remove sediment Small biomass quantities
	Treatment with detergents		Isolates conductive structures Produces clean samples Allows for a range of chemical treatments (e.g. SDS and EDTA)	Biased towards larger/more robust species Time-consuming Precise incubation times must be kept
	Extracting via centrifuge		Easy to do Produces larger biomass samples than picking May be combined with treatments such as SDS	Sediment still present May damage the filaments
Morphology	AFM		High-resolution information on surface structure and forces Allows for conductivity measurements Samples can be imaged in water Non-destructive Tip can be used to alter cells at nanoscale	Challenging on larger samples/structures Lower lateral resolution than SEM Long acquisition times Few cells can be imaged at a time
	SEM	CB filament needs to be sputter coated with conductive metal	Provides topological information from electron density Better lateral resolution than AFM Possible to use with 3D structures Can analyse chemical composition at the surface if combined with EDX Multiple filaments can be analysed in one session Nanoscale imaging	Low height resolution Destructive to samples due to ion beam
	TEM	Samples must be thin (∼ 200 nm)	Provides information on internal structure at nanoscale resolution Can build 3D models of internal structure (Cryo-EM)	
Electrochemical and electrical properties	Cyclic voltammetry		Can be done in sediment or with filaments on an electrode Provides information on redox properties Can be done with native filaments and extracted fibre skeletons	Hard to isolate CB redox peaks from sediment Requires anoxic environment
	Conductivity measurements		Provides insight into the mechanism of conductivity Can be done with native filaments and extracted fibre skeletons	Requires anoxic environment
	Chemical analyses (Raman, XRM, XRF)		Shows chemical composition and structure of conductive fibres	Requires extracted fibre skeletons Often necessitates nanoscale equipment due to low biomass
Physiology	Meta-transcriptomics, proteomics, metabolomics	Bulk-sediment sample	Provides insights into the expression of genes, rather than just presence Potential to determine the proteins involved in conduction mechanism Can analyse multiple species simultaneously	Not implemented broadly yet in the field Can be challenging to acquire high quality results/data Challenging to interpret data from highly complex environments (such as sediment)
	nanoSIMS and stable isotope labelling		Provides information on the growth of individual cells	Can be expensive due to isotopes required Cannot be done *in situ* CB (for current experimental designs)
	Planar optodes		Works with CB *in situ*	Does not give information on growth of individual CB or cells
Motility	Trench slides and microscopy		Allows for direct observation of CB Can be coupled with other techniques such as planar optodes or Raman microscopy	Difficult to standardize
	Agar columns/tubes		Could allow for the testing of artificial concentration gradients	Largely untested
Diversity	Microscopy	Picked filaments	Easy to do with a wide range of methods (FISH, AFM, SEM, TEM, etc.) CB have distinct morphological features	Several species may have similar features, limiting the accuracy of these observations
	DNA/RNA sequencing	Either single strain incubation, or extracted filament	Accurate at distinguishing between species Allows for official validation of species for single-species culture Allows the study of population dynamics	Challenging to get high-quality sequences
	Amplicon sequencing (DNA or RNA)	Core slicing is needed	Commercial amplification kits and protocols work well with CB Serves to assess both presence (DNA) and activity (RNA) of CB in the same sample Amplicon sequencing provides information about the entire microbial community Targets 16S gene Allows determination of diversity of CB in a sample (important for single-species cultures)	Less efficient for CB compared to other bacteria due to rigid cell envelope Amplicon sequencing provides relative abundance only Destructive method May also detect DNA from (recently) dead CB
	Whole genome assembly		Allows for whole genetic analyses Provides higher resolution of taxonomy	Requires high-quality sequencing Challenging to accomplish
	DNA extraction for (meta)genomics	CB from the environment, enrichment culture, or picked filaments can be used	Can compare genes from CB to other species to understand their function Has the potential to be combined with non-destructive imaging techniques like AFM	Can give results of varying quality depending on relative abundance, strain heterogeneity, and technique CB have high proportion of unknown genes in part, due to their unique metabolism Destructive method

## Culturing

It is currently thought that the lack of successful cultures in growth media likely arises from an incomplete understanding of the metabolism of CB and their requirements for microbial co-community both in symbiosis and possible predation (Stewart 2012, Vasquez-Cardenas et al. 2015, Thorup et al. 2021, Liau et al. 2022, Bjerg et al. 2023, Lustermans et al. 2023). This leads to the current approaches, where CB can be cultured relatively easily in incubated sediments, but not in any other media [with notable, recent exceptions of agar pillars (Sachs et al. 2022) and the first reported case of success with artificial sediment (Stiefelmaier et al. 2024)].

### Enrichment cultures in natural sediment

**Figure 3.**
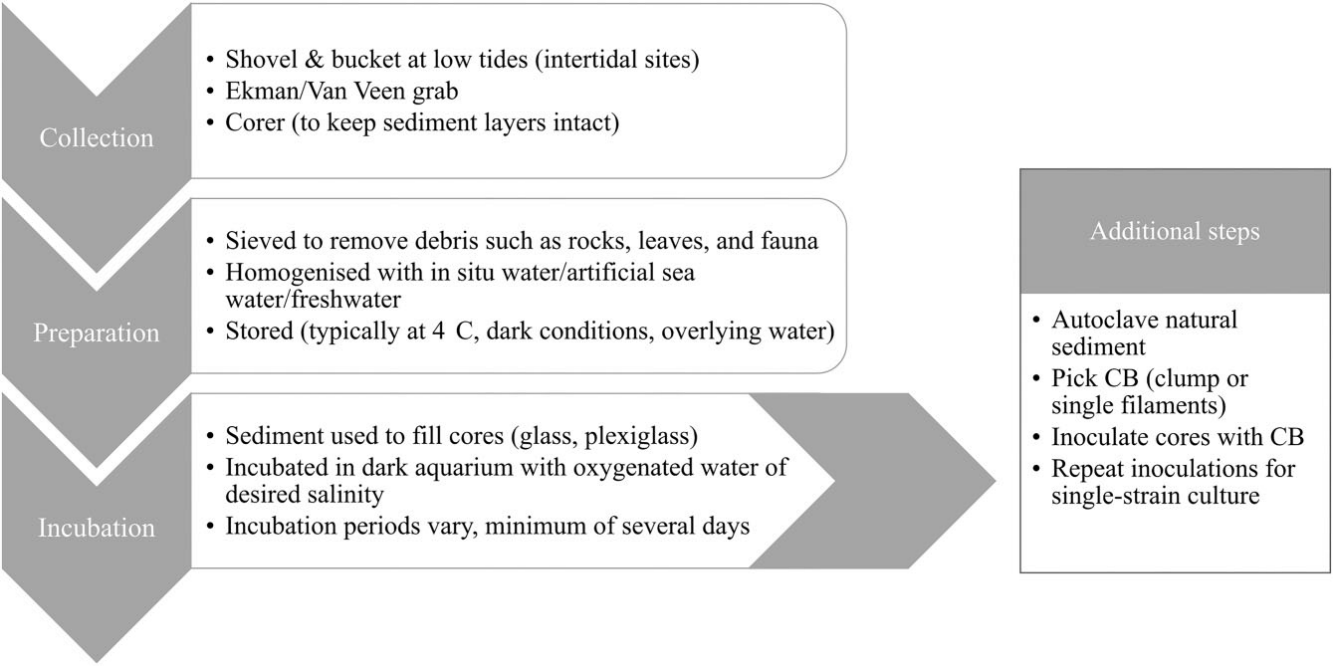
The key steps involved in sediment collection, preparation, and incubation for creating CB enrichment cultures. There are multiple options for each step, which can be chosen based on field site and sediment characteristics and the type of enrichment culture needed for experiments.

The first step to making an enrichment culture of CB is the collection of sediment (top 20 cm) from a field site. CB are distributed worldwide, and are commonly found in sediments with favourable conditions, such as high sulfide concentrations, minimal bioturbation, and small grain size (Malkin et al. 2014, Burdorf et al. 2017). Figure 3 outlines the general procedure and options for sediment collection and incubation preparations.

Bulk sediments can be immediately stored, but are commonly sieved to remove debris and fauna that may interfere with experiments; however, this could shorten CB filament lengths leading to an increase in incubation time. Sediment is generally stored at 4°C, ideally in the dark and with overlying water, for extended periods of time. Whilst the effects of temperature on CB are currently unknown, success has been reported with sediment stored for several months (Hermans et al. 2020), and potentially years with anecdotal observations across multiple labs.

Homogenizing sediment with artificial seawater prior to core preparation may be beneficial to ensure porewater salinity matches that of the aquarium. After letting the sediment settle, excess water can be removed and used to fill the aquarium. Having consistent salinity is important in determining which species of CB and other microbes are dominant in a system (Dam et al. 2021).

Cores can then be prepared from the sieved, homogenized sediment, and incubated in an aquarium bubbled with air to maintain oxygenation, and made to the desired experimental salinity. Sediment is left to settle in the cores for 12–24 h, after which the surface can be levelled by pushing the sediment up a few millimetres in the core. Incubation times in the literature vary from 10 days (Schauer et al. 2014), up to several weeks or months (Geerlings et al. 2021, Vasquez-Cardenas et al. 2022, Yin et al. 2022). Incubation time allows the CB to colonize the sediment, with e-SOX fingerprint (Fig. 5) typically reforming within several days (Schauer et al. 2014). CB are likely active well before 10 days, but may be shorter in length. In contrast, longer incubations show a decrease in CB activity after day 120 (Hermans et al. 2020). It is important to monitor salinity throughout incubation periods, as it can vary due to evaporation, and changes (±5 units) will affect the microbial community, particularly in favouring the development of one CB species over others (Dam et al. 2021).

Whilst this method (Fig. 3) has been shown to have a high chance of success when done with sediment from a CB-rich site (Burdorf et al. 2017), it does result in a culture containing many species of both CB themselves and a variable range of microbes from the environment (Marzocchi et al. 2014, Dam et al. 2021). Depending on the research purposes, the complexity of the microbial community may not be an issue, but when trying to isolate effects due to CB, the high degree of environmental variability can pose a challenge in distinguishing CB effects from those created by other variations in microbiome.

### Enrichment cultures in autoclaved sediment

**Figure 4.**
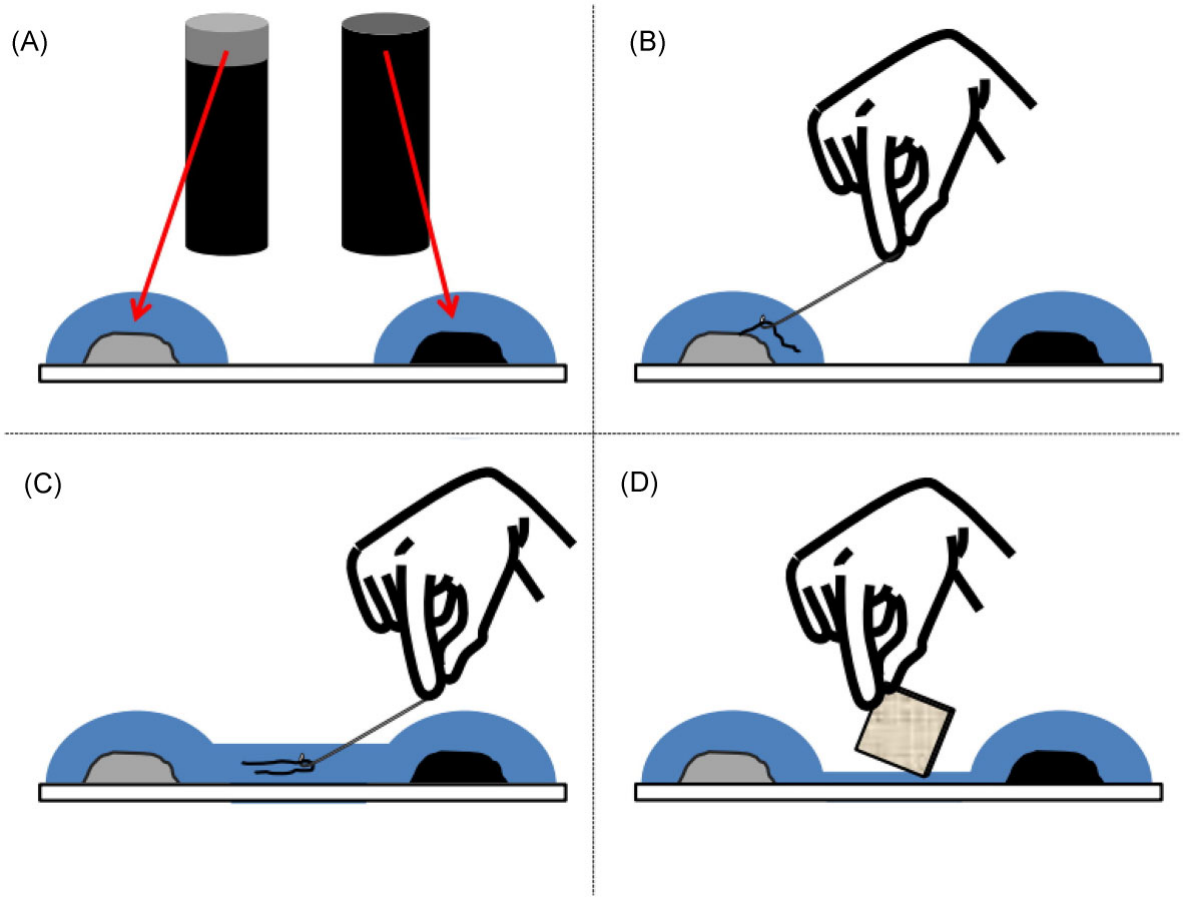
Schematic showing the steps to inoculate autoclaved sediment with a singular CB, creating a single-species culture. This involves taking sediment from an autoclaved core and an active core (A), picking a cable bacterium (B), and placing it into the autoclaved sediment (C), then removing the water between the two (D), following which autoclaved sediment is placed into a core (Thorup et al. 2021). Reproduced with permission from Elsevier Publishing under CC BY 4.0 licensing.

One method that has been developed to limit the variability of microbial communities in enrichment cultures is the use of autoclaved sediment (Thorup et al. 2021, Li et al. 2022). Enrichment cultures as described above are used to inoculate autoclaved sediment with CB. To do this, natural sediment and (sea)water (or artificial seawater) are autoclaved at 120°C for 0.5–2 h and cooled (although the temperature and time used in previous studies have varied). Autoclaved sediment can be stored refrigerated for several weeks, or used immediately to fill cores, which are then inoculated with live CB (and any adhered microorganisms) from an enrichment culture in natural sediment (Fig. 4, the ‘Picking native filaments’ section). Manure or other sources of microbes can be added to the autoclaved sediment to provide a more complex microbiome (without CB) whilst keeping variation between treatments minimal (Scholz et al. 2020). Overall, autoclaved sediment reduces the complexity of the system compared to natural sediments.

Autoclaving sediment can affect the geochemistry of sediments due to the constant, high temperature. In particular, autoclaved sediments can contain low sulfide concentrations resulting from the high temperatures and exposure to oxygen (Li et al. 2022), limiting CB growth. This can be remedied by adding a source of sulfide such as iron sulfide, but as a wide range of characteristics such as pH and organic carbon will be altered, it is important to determine their effects if they are relevant to the experimental outcomes (Li et al. 2022). It is also important to note that if sediment is autoclaved more than once, inoculation of CB is generally unsuccessful. This may be due to changes in sediment composition or structure, or microbes (such as fungi) surviving initial autoclaving being necessary for CB growth (Thorup et al. 2021); however, there is still not much knowledge of the interactions between CB and the microbiome, so there may be more work to understand their potential effects.

### Single-species cultures

A common challenge with both of the culturing approaches described above is in studying the physiological and geochemical effects of individual species as opposed to the CB community more generally given the diversity found within the CB clade. Although one species of CB can dominate an environment (Liau et al. 2022), there are often several species and/or strains of CB present in a given sediment (Marzocchi et al. 2014, Trojan et al. 2016, Dam et al. 2021, Geelhoed et al. 2023, Plum-Jensen et al. 2024). To avoid this, a method was developed to grow cultures with a single strain of freshwater CB, *Electronema aureum*, which was the first fully characterized single-species culture (Thorup et al. 2021). More recent success has been observed with marine and estuarine species *Electrothrix aestuarii (scaldis)* (Plum-Jensen et al. 2024).

To attain a single-species culture, a single filament is taken from an existing enrichment culture and transferred into a core with autoclaved sediment. The filament must be rinsed in filtered (sea)water on a microscopy slide and kept submerged throughout this process to increase its viability following the transfer (Fig. 4) (Thorup et al. 2021). Autoclaved sediment cores with one CB filament are then incubated for at least 3–4 weeks until filaments can be observed in the sediment microscopically (Thorup et al. 2021, Plum-Jensen et al. 2024).

In these successful studies, the process of single filament transfer was repeated until the growth of a single species was confirmed via amplicon sequencing (the ‘16S rRNA gene’ section), after which clumps of CB-rich sediment were transferred. Starting a culture with a single filament is not only more time-consuming, but it has a low success rate of ∼20% (Thorup et al. 2021). In contrast, inoculation using a clump of sediment allows for a better chance of success in growing CB (≈100%, compared to 21% with single filaments) (Thorup et al. 2021), but if done with sediment containing multiple species, it would not result in a clonal CB culture. As a result, it is important to consider the necessity for a genetically homogenous culture and balance that with time constraints to determine which inoculation method is most appropriate.

An aspect of single-strain culturing that remains somewhat unclear is what characteristics of these particular species allow them to grow and thrive in long-term enrichments, where others fail. The increase in described species from *Electronema aureum* to include multiple *Electrothrix* species indicates that success may be in part due to the work from researchers to create and care for ongoing cultures. However, these species may also contain adaptations that make them more resilient to being extracted from sediment and transferred to a new environment. There may also be variation between species that provide them more resistant to potential predation within the sediment, allowing them to survive longer in enrichment cultures than other species.

In maintaining ongoing cultures, it has been observed that the activity of CB in any single core (natural or autoclaved sediment) will reach a peak and then level off over time (Malkin et al. 2014, Schauer et al. 2014). The reasons behind this eventual decline are not fully understood but could be a combination of factors such as nutrient availability, degradation of organic matter, depletion of FeS, or competition from other microbes (Seitaj et al. 2015, Meysman 2018). CB may be susceptible to viral (Kjeldsen et al. 2019), providing a possible hypothesis as to why CB numbers decline over long time periods. To circumvent this, new cores can be made every few weeks (Thorup et al. 2021) to provide consistent access to active CB cultures.

### Negative controls

When designing experiments investigating the effects of CB on environmental variables (e.g. solute concentrations, gas emissions, microbiomes), negative controls are needed. These cores need to have the same biogeochemical characteristics as the positive controls, but without active CB present. In the previous section, the challenges in growing CB were discussed; however, preventing them from growing is equally challenging. Nevertheless, three approaches are used: autoclaved sediment, severing the flow of electrons, and inhibiting CB growth.

Using autoclaved sediment cores in parallel has not been commonly applied due to the impact of autoclaving on biogeochemistry, but by incubating cores with and without added CB, differences may be observed (Thorup et al. 2021).

More commonly, severing the electrical transport is done by passing a thin metal or nylon wire through the sediment just below the oxic zone (Pfeffer et al. 2012, Vasquez-Cardenas et al. 2015). This cut slices CB filaments in two, effectively impeding the transfer of electrons along their filaments (Pfeffer et al. 2012). Whilst the effect is immediate, it is only effective for short time frames as CB can both migrate and regrow (this allows culturing in cores made of homogenized sediment possible) (Bjerg et al. 2016, Malkin et al. 2017), and efficiently recolonize sediment layers. The return of the e-SOX fingerprint can take place within 24 h if the cut is shallow (<5 mm) but possibly longer with slightly deeper cuts (Vasquez-Cardenas et al. 2015). Overall, this makes the physical severing of LDET useful for experiments with short timeframes only, as long-term experiments require frequent cutting of the sediment, which may disturb the core and potentially create a treatment artefact.

Placing polycarbonate filters at a depth of <1 cm in a sediment core is another way to prevent the growth of CB in natural sediments. This was shown to prevent the appearance of a surface pH peak over 20 days, which indicates an absence of CB activity (Pfeffer et al. 2012). The filter acts as a physical barrier between the oxic and anoxic layers, which prevents CB from being able to grow and access both zones in the sediment. The porosity of the filter does allow smaller particles and solutes to diffuse unimpeded throughout the sediment column and porewater. It is therefore important to consider the pore size of the filters, with <0.8 µm being recommended, whilst pore sizes of 2 µm or larger do not prevent the growth and migration of CB (Pfeffer et al. 2012). Filters should be placed just below the oxic zone ∼3 mm to effectively limit growth, but some studies have placed filters at 10 mm with success (Yin et al. 2022).

Interestingly, CB have been observed at depths below the filter paper, but may have been inactive or active using nitrate as an electron acceptor for their metabolism (Marzocchi et al. 2014). This again highlights the difficulty in making adequate negative controls where CB are entirely absent.

### Comparison of existing methods and alternatives

Each of the methods described above for preparing cultures of CB has merits and drawbacks (Table 1), which should be considered when designing experiments. For example, native sediment is preferable for biogeochemical experiments due to the impact of autoclaving on the variables being measured. This holds true both when looking at the effects of CB on the environment, as well as investigating the effects of additives (such as sulfide or nitrate) on CB populations (Marzocchi et al. 2014, Xu et al. 2021a,b). In these examples, negative controls without CB are generally required when looking at the effect of e-SOx on the environment, and the specific approach taken may depend on how the sediment core is analysed later on, as filters may impede microelectrode profiling, e.g. (the ‘Activity’ section) (Kessler et al. 2018).

Single-species cores, whilst more challenging to create, are valuable for studying the genomics of CB species as they facilitate high-quality sequencing (Hiralal et al. 2024a,b). However, it is also possible to perform metagenomic studies from sediment with complex microbial communities (Sereika et al. 2023), highlighting the value in having a range of approaches.

There are also indications that in the future, CB culturing may not be limited to sediment. It has been shown that CB can attach to electrodes, and may be capable of using these as electron acceptors (Reimers et al. 2017, Li et al. 2020, Bonne et al. 2024), providing an alternative pathway to creating more dense CB cultures. Using electrodes in growing CB cultures could aid in concentrating CB filaments to a surface, making extraction less time-consuming.

Another novel technique is the use of agar pillars in the middle of the sediment core (Sachs et al. 2022). Due to the porous nature of agar, the diffusion of solutes and migration of microbes from the surrounding matrix is possible. There were some differences noted such as deeper oxygen penetration in agar than sediment, but notably, CB were identified within the agar as well as sediment. As a result, this may be a useful technique to create freshwater enrichment cultures with fewer of the complex interactions that make working with sediment challenging.

Determining the minimum requirements for CB to grow is needed to culture them in a lab environment. The discovery that they can thrive in autoclaved sediment and can grow attached to electrodes when oxygen is absent gives some clues as to what CB need to survive, but to create a simpler, axenic culture, more work needs to be done in this area. Very recently a study showed success in cultivating CB in synthetic sediment, providing a crucial step forward in this area (Stiefelmaier et al. 2024). Through the careful curation of solid components, nutrients, and microbiome, the use of artificial sediment may allow for enhanced control over environmental factors in future experimental designs. This may also address one of the drawbacks of using native sediments, which is that growth conditions vary significantly across sites and experiments, making comparison between some studies somewhat fraught.

These new and innovative approaches require further investigation to determine their suitability for ongoing cultures, as well as for sustaining different species of CB. In particular, there has been more success cultivating freshwater CB in unique conditions, and it is important to avoid potential bias away from marine species as the field continues to develop (Thorup et al. 2021, Sachs et al. 2022, Bonne et al. 2024, Stiefelmaier et al. 2024).

## Detection

As sediment is opaque, CB cannot be observed easily in the field, but there are visual indicators that they may be present such as enriched iron oxides (light brown or orange), sulfide depleted (grey), and carbonate layers in a sediment core, which can be observed without disruption to the sediment (Risgaard-Petersen et al. 2012). However, a range of other methods have been developed to more accurately detect and quantify these bacteria.

An important aspect of quantifying the abundance of CB compared to non-conductive microorganisms is the difference between electrical activity and density of filaments in the sediment. Many of the methods that will be discussed measure the electrical activity of CB, essentially only detecting those that are conducting electrons at the time of measurement. This does not include CB that are dormant, do not have access to oxygen, or have died. In contrast, both active and inactive CB (to a degree) can be detected with methods that measure the actual biomass of CB within the sediment. Whilst the activity of CB can be measured non-destructively using microprofiling, density measurements typically require sacrificing the core for extractions.

### Activity

**Figure 5.**
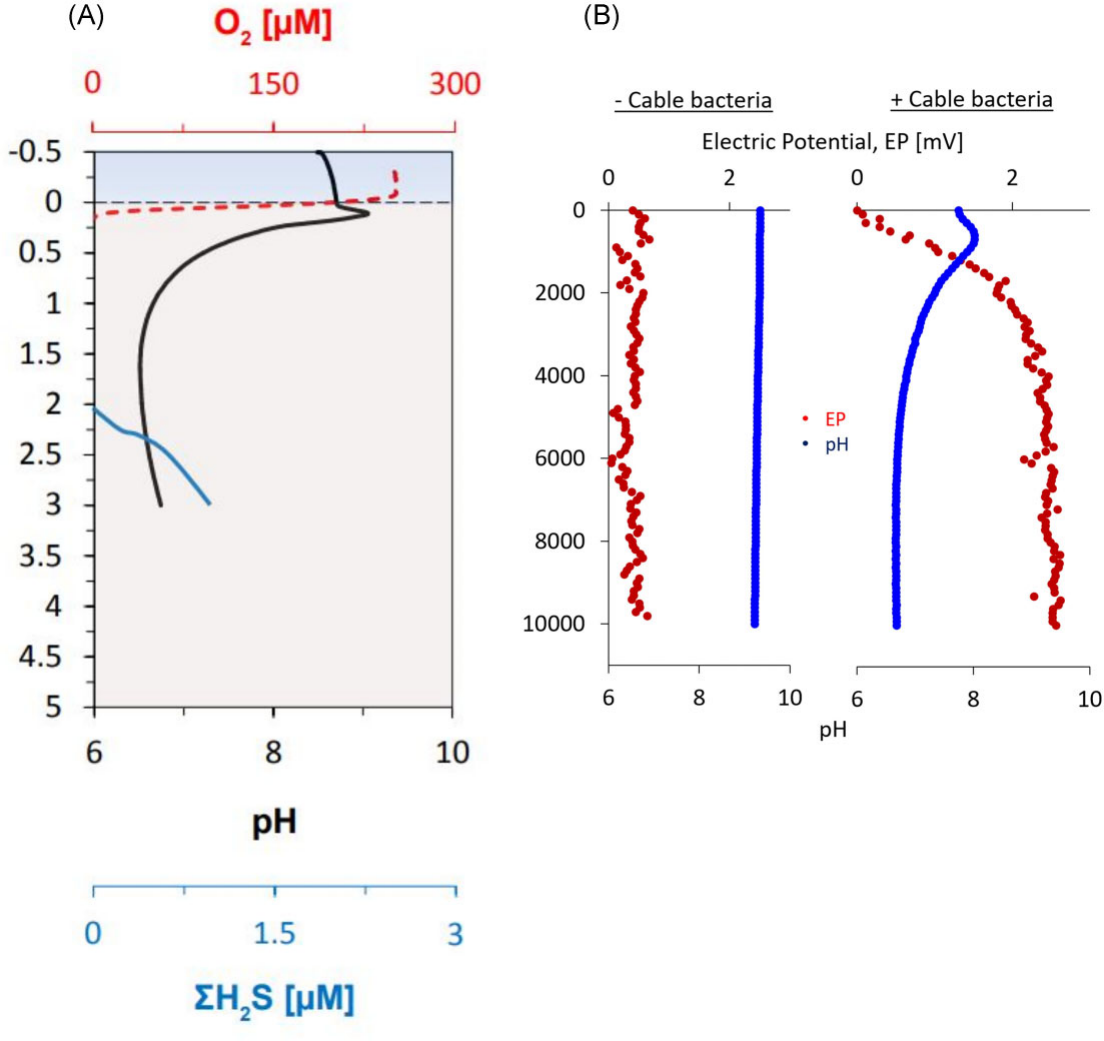
(A) Profiles of pH, O_2_, and H_2_S in sediment with an active CB population (Hermans et al. 2020). Reproduced with permission from Copernicus Publications under CC BY 4.0 licensing. (B) Electrical potential profiles of a core with (right) and without (left) active CB, showing the increase in EP from the surface to greater depths (dotted profile) (Thorup et al. 2021). Reproduced with permission from Elsevier Publishing under CC BY 4.0 licensing.

The activity of CB was first identified by the ‘geochemical fingerprint’ they create in sediment (Fig. 5A) (Nielsen et al. 2010, 2015, Meysman et al. 2015). The distinguishing feature is the pH peak in the oxic zone, and acidification of deeper sediment. In the absence of benthic photosynthetic activity, which can create a similar pattern in pH (Revsbech and Jorgensen 1983), this can be used to determine the presence of CB in sediment. In addition, the consumption of sulfide by CB creates a suboxic zone devoid of oxygen and sulfide, making these profiles additionally useful in indicating their presence as seen in Fig. 5A. This unique geochemical fingerprint can be efficiently identified both *in situ* and enrichment cultures using microelectrode profiling of pH, O_2_, and H_2_S with existing equipment (Nielsen et al. 2010, Pfeffer et al. 2012, Malkin et al. 2014). By using these three parameters (pH, O_2_, and H_2_S), both the cathodic consumption of oxygen and anodic consumption of sulfide can be calculated (Nielsen et al. 2010, Risgaard-Petersen et al. 2012, Malkin et al. 2014, Müller et al. 2016). These rates can be used to estimate the current density produced across the sediment core, which can be used to compare activity between sites and treatments.

A more direct estimation of current density is obtained from electric potential (EP) profiling. EP microelectrodes were developed, in part due to the discovery of CB, to facilitate investigation of the electrical characteristics of sediment at microscale resolution (Damgaard et al. 2014). These electrodes have the same advantages as other microprofiling techniques discussed previously, with minimal disturbance of sediment due to the micron-sized tip (50–200 μm), allowing for repeat measurements of a single core. A typical EP profile of sediment with active CB is shown in Fig. 5B, with a low EP at the surface, increasing across the oxic–anoxic border, then plateauing at greater depths.

EP microelectrodes work by measuring the electrical potential at the location of the tip of the electrode relative to a reference electrode placed in the overlying water. To ensure that redox conditions do not impact the readings, they are typically made with Ag/AgCl half cells, which are located further up the body of the electrode with electrolyte bridging the path to the opening. This makes them highly accurate in measuring EP, as redox potentials could otherwise create false EP readings.

When EP measurements are used to measure the activity of a CB population, it is important to consider and maintain the salinity of the system. Freshwater systems tend to give clearer EP measurements because lower conductivity in porewater leads to higher EP readings for the same rate of CB activity. In freshwater samples, the maximum EP seen when CB are present can be >30 mV (Risgaard-Petersen et al. 2015), whilst brackish and marine samples are often <2 mV (Hermans et al. 2020). In addition, EP is highly sensitive to salinity gradients as the diffusion of ions can create an electrical field, confounding the CB-induced EP gradients. If the salinity of overlying water is mismatched with porewater salinity, the EP profiles will show a gradient not related to the activity of CB (Damgaard et al. 2014). However, despite this additional challenge, EP measurements remain one of the easiest and most accurate methods of determining the activity of CB in sediment.

An advantage of EP profiling is that it can also be used to calculate current density, and provides a more accurate measurement than using O_2_ and H_2_S fluxes (Risgaard-Petersen et al. 2014). To do this, current density is calculated from the gradient of the EP profile across the oxic–anoxic interface multiplied by the sediment conductivity. This provides an estimate of the CB actively conducting electrons at the time of measurement. Initially, high-resolution sediment conductivity measurements were done alongside EP profiles by incorporating sediment cores into electric circuits to induce a current. Then the EP could be compared with and without the additional current to find the baseline conductivity and determine if it changes through the core (Risgaard-Petersen et al. 2014). Due to the extra equipment needed to do this, it is not often done but would be highly recommended in cases where determining porewater conductivity and sediment porosity is not straightforward.

Taking the calculations from EP profiling further, the rate of reactions at the anodic or cathodic end can be calculated using general mass diffusion models, e.g. in the software PROFILE (Berg et al. 1998, van de Velde et al. 2016). Using EP profiles and conductivity in the place of concentration profiles and diffusion coefficients, respectively, PROFILE allows for the calculation of rates of consumption/production of electrons, from which *A*/*m*^3^ can be calculated for a sediment profile. The advantage of this over just determining current density is that these calculations can show not only quantitative activity of CB, but also the depths at which they are producing or consuming electrons.

Overall, these advantages make EP microelectrode profiling the dominant technique for measuring the activity of CB in a sediment core. As it is a non-destructive method, it allows for long-term experiments such as understanding how CB activity develops over time under different conditions (Hermans et al. 2020). However, challenges include interference from salinity gradients and sensitivity limitations. Particularly when measuring small changes in EP (such as those seen in marine samples), noise can pose an issue due to EP being sensitive to electrical interference coming from other equipment, static, or insufficient grounding. In addition, microelectrodes are very delicate and can be easily damaged or broken, even in the middle of a profile, making large-scale experiments potentially expensive.

### Density

#### Fluorescence in situ hybridization

The main alternative to detecting CB through EP is using cell/filament quantification to determine abundance or density of the CB biomass. Methods used to measure density are typically destructive to the sediment core but allow for direct measurements of CB abundances through counting or other forms of quantification. This means that these methods are challenging to apply in long-term incubations (require many parallel cores), so they are often only undertaken at the end of an experiment/incubation period to get a measurement of final density. The most common method used is fluorescence *in situ* hybridization (FISH), performed on a sediment sample.

Initially developed in the 1980s (Bauman et al. 1980, Levsky and Singer 2003) to visualize nucleic acids, FISH has had many iterations and modifications to improve and broaden the range of applications over the intervening years. In FISH, a fluorophore is attached to a DNA or RNA string complementary to a sequence in the region of interest within a cell. Then the DNA/RNA within the cell is pulled apart through chemical treatment and the fluorescent string bound onto it. This allowed for the targeting of specific species, or even chromosomes within cells for fluorescence (Pernthaler et al. 2001, Levsky and Singer 2003).

In the context of CB research, the fluorescent-labelled probes target a region of the 16S rRNA gene that is specific to the phylogenetic clade of interest (Trojan et al. 2016). Formamide is used in the hybridization process to open the rRNA strand within the cells, and must be optimized for each probe. The probe DSB706 targets the family Desulfobulbaceae (Loy et al. 2002, Schauer et al. 2014), of which CB are a subgroup, and is commonly used for quantification with formamide concentrations of 45% (Li et al. 2020), compared to 35% for EUB338 (positive control) and NONEUB (negative control) (Schauer et al. 2014).

As DSB706 targets a range of microorganisms, the morphology of CB is also used to aid in identification, and their length can be measured (Pfeffer et al. 2012, Schauer et al. 2014, Seitaj et al. 2015, Sulu-Gambari et al. 2016). Whilst DSB706 remains the most common, more recently a wider range of probes have been developed (Table 2) to distinguish between different species of CB (Trojan et al. 2016, Geelhoed et al. 2023, Sereika et al. 2023), which allows for the quantification of individual CB species in the same sample.

**Table 2.** FISH probes targeting the 16S rRNA gene of CB.

Probe	Sequence (5′–3′)	Target specificity	Helper/competitor probes	% FA	Reference
EXaa430	TTT CTT CCC TTC TGA CAG GGT TT	*Electrothrix arhusiensis* **AR5** (Geelhoed et al. 2023)	H409^a^ C432^b^ C444^b^	45	Trojan et al. (2016)
EXco1016	CTC TCA AAG AGA GCA CTT CCC TA	*Electrothrix communis* *Ca*. Electrothrix japonica ***Electrothrix laxa*** (Sereika et al. 2023) **AR1** (Geelhoed et al. 2023) **AR5** (Geelhoed et al. 2023) **MAN1_4** (Geelhoed et al. 2023)	H994^a^	45	Trojan et al. (2016)
EXma1271	GCT TTC AGG GAT TTG CGC CT	*Ca*. Electrothrix marina	H1229^a^ H1251^a^	45	Trojan et al. (2016)
EX-lin-189	CCG CCT TTC TTG ATC GCC CTT	***Electrothrix laxa*** (Sereika et al. 2023)	EX-lin-189_C1^b^	30	Sereika et al. (2023)
Egiga134	CTA TCC CGA GCA TCT GGA	*Electrothrix gigas*	/	35	Geelhoed et al. (2023)
ENni1437	CCC GAA GGT CCG CCC AGC T	*Electronema aureum* *Ca*. Electronema nielsenii	/	50	Trojan et al. (2016)
ENpa1421c	CCA GCT GCT TCT GGT GCA ATC G	*Ca*. Electronema palustre	/	45	Trojan et al. (2016)
EN-logt-80	CGC CAC TTT CGA TTC TCC GAA GAA	*Electronema halotolerans*	/	35	Sereika et al. (2023)
FliDSB194	GGA GAG GTC TCC TTT CCT TA	Groundwater cable bacteria	/	35	Müller et al. (2016)
DSB706	ACC CGT ATT CCT CCC GAT	Desulfobulbaceae	/	35	Loy et al. (2002)

Species indicated in bold were not originally targeted when the probe was designed. % FA = formamide concentration (v/v) of FISH hybridization buffer at 46°C.

a Helper probes.

b Competitor probes.

**Figure 6.**
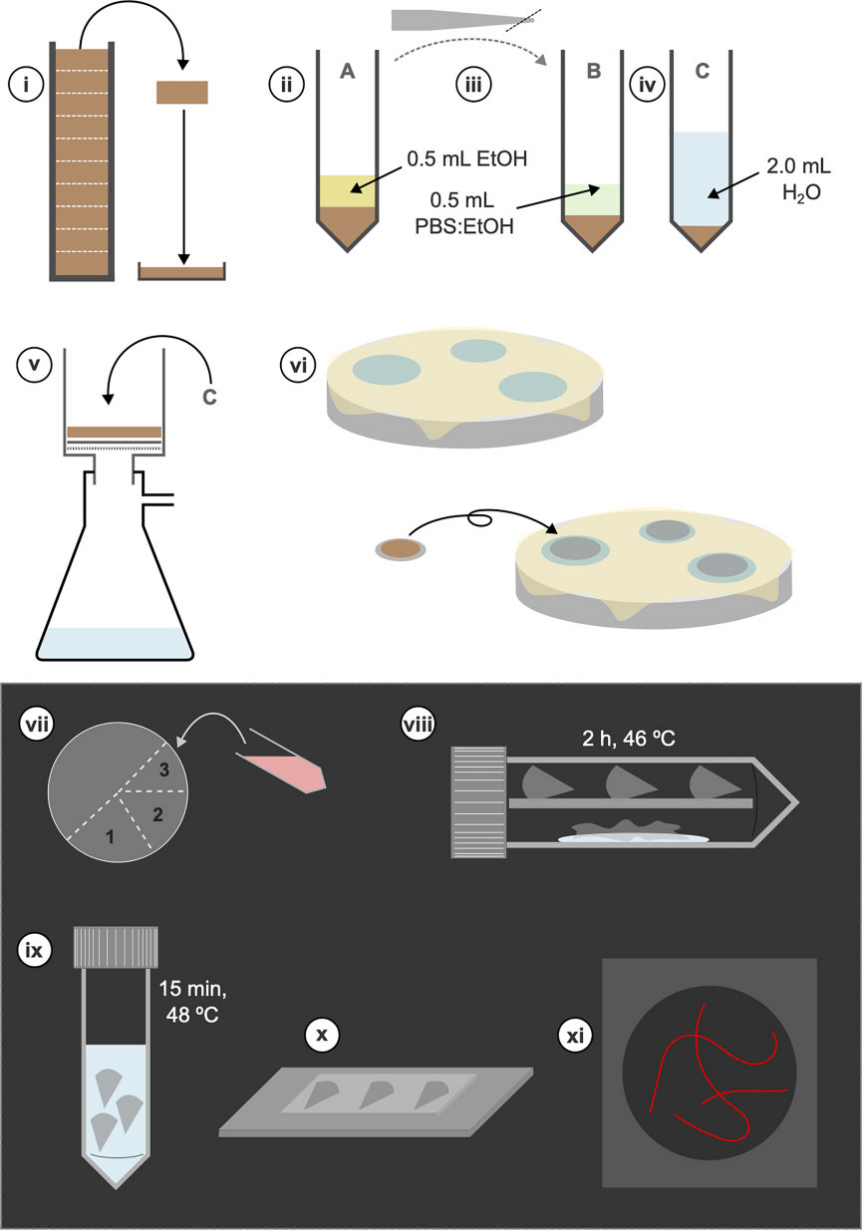
General protocol for performing FISH for CB filament quantification. The steps shown here are (i) slicing core into segments as required and homogenizing each sediment sample. (ii) Preserving sediment in ethanol. (iii) Preparing a pipette tip by slicing the end to minimize clogging when transferring sediment samples. (iv) Diluting sediment in appropriate solutions. (v) Filtering the diluted sample (sediment in water) onto a 0.2 µm polycarbonate filter with a membrane support filter (0.45 µm). (vi) Coat the filter in agar by briefly placing it sample-side-up on an agar drop and then letting it dry sample-side-down on an agar drop overnight; filters can be kept frozen at this stage for preservation. (vii) In the dark, the probe made up with hybridization buffer is added to the sample filter, with other sections kept for positive and negative controls. (viii) Filter-probe samples are incubated for 2 h at 46°C in a sealed tube with the excess buffer used to maintain humidity. (ix) Filter-probe samples are placed in a washing buffer for 15 min at 48°C. (x) Filters are placed onto a slide with a mounting medium. (xi) Microscopy images are then used to calculate the density of cable filaments in the initial sediment sample.

The procedure for FISH to quantify CB density in sediment is outlined in Fig. 6, typically giving results in the m/cm^3^ range (Schauer et al. 2014). Sample fixation is performed with a 1:1 ratio of sediment to ethanol (Schauer et al. 2014), allowing for storage over longer time periods. There are several alternatives for sample preparation such as washing sediment to extract CB bundles to the supernatant, which, whilst less common, may improve accuracy of cell counts by better separating CB from sediment particles (Müller et al. 2016, Liau et al. 2022).

The advantage of FISH is that it distinguishes CB from other filamentous organisms such as algae. However, variability in calculated densities of CB can be quite high (Risgaard-Petersen et al. 2015, Geelhoed et al. 2020), but the rapidly improving probe design may reduce this over time. The limit of detection has been estimated at 1.5 × 10^6^ cells cm^−3^ and 10^4^ filaments cm^−3^ (Schauer et al. 2014), which many enrichment cultures exceed, making FISH a valuable tool in comparing CB densities between sites and treatments.

#### Amplicon sequencing and qPCR

The main alternative to FISH in quantifying CB abundances is in measuring the DNA or RNA makeup of a sediment sample. Initially, 16S cDNA clone libraries were used to obtain genetic information from CB and give insights into the relative abundances of their surrounding microbial communities (Malkin et al. 2014, Vasquez-Cardenas et al. 2015). Currently, CB quantity is assessed by amplicon sequencing (see the ‘16S rRNA gene’ section) and quantitative polymerase chain reaction (qPCR) (qPCR). Whilst 16S rRNA gene amplicon sequencing does not provide absolute abundances of bacteria, it reveals their relative abundances within a microbial community. Different regions of the 16S rRNA gene have been targeted, but the V3-V4 region has been the most widely used (Dam et al. 2021, Scholz et al. 2021, Thorup et al. 2021, Vasquez-Cardenas et al. 2022). Both DNA and RNA extracted from time series CB enrichments (V4-V5 region of the 16S gene) found surprisingly high (up to 65%) relative CB abundances among the RNA amplicons, corresponding to RNA:DNA ratios of 20–30 (Liau et al. 2022). This was attributed to the fast growth rates of these bacteria and agrees with the observation that CB can dominate the microbial metabolism in sediments (Risgaard-Petersen et al. 2014, Liau et al. 2022).

Next to amplicon sequencing, qPCR was established as a method to obtain absolute (and relative) abundances of CB against a standard curve, with results being reported as target gene copy numbers per gram of wet sediment (Geelhoed et al. 2023). In a study by Geelhoed et al. (2020), a qPCR protocol was presented in which three different primer pairs were used to target the 16S rRNA gene of total Eubacteria, the family Desulfobulbaceae, and four *Electrothrix* species (*arhusiensis, communis, japonica*, and *marina*). A qPCR protocol targeting single-strain cultures of *Electronema aureum* has been published recently (Bonne et al. 2024).

As an alternative target to the 16S rRNA gene, Liu et al. (2021) presented a qPCR protocol targeting CB using the *dsrB* gene, a common phylogenetic marker gene for sulfate-reducing and sulfide-oxidizing prokaryotes, encoding for the dissimilatory sulfite reductase (Müller et al. 2015, Trojan et al. 2016). Primers were designed based on 21 *dsrB* sequences of the Desulfobulbaceae family, containing 9 *Electrothrix*, 2 *Electronema*, and 10 sequences from sister genera of CB (Liu et al. 2021).

With specific primers, qPCR is a fast method to estimate CB abundances. However, compared to FISH, the requirement of target-specific primers may bias quantification if there are unidentified species or genera of CB present in a sample. Whilst relative abundances can be inferred from amplicon sequencing data, qPCR and FISH allow for the determination of absolute abundances.

### Detection: activity and density summary

When measuring the activity of CB in a sediment core, EP profiling tends to be the main *in situ*, non-destructive technique providing an accurate measure of activity. Whilst other microelectrode depth profiles (such as pH or H_2_S) can indicate the presence or absence of CB, using pH, O_2_, and H_2_S depth profiles to calculate current density is likely only accurate on natural sediments due to the assumptions made in the calculations. For example, low sulfide in autoclaved sediment may render sulfide profiles less useful in accurately measuring CB activity. This makes EP the dominant method thus far, allowing activity results to be highly comparable across the field, as many previous studies have used EP profiles to estimate current density as an indicator of CB activity (Risgaard-Petersen et al. 2015, Burdorf et al. 2016, Marzocchi et al. 2018, Hermans et al. 2020, Dam et al. 2021, Lustermans et al. 2023). On the other hand, in conducting robust research, it is important to have other methods to check results against, and pH/H_2_S profiles are indispensable to estimate the maximum depth of CB growth. There is currently a gap in the field for other ways to assess the number of active CB within sediment cores over time. Using more replicates to take FISH or qPCR samples at various timepoints is a possible solution (Burdorf et al. 2018); however, they may also include inactive and recently dead CB biomass. There may also be other proxies such as isotope analysis or iron precipitation rates that could provide yet more estimates of CB activity based on concentration or reaction rates. Ultimately, direct measurements require the extraction of all CB filaments from the environment, which currently has not been achieved from cultures or *in situ* samples.

In contrast, FISH, qPCR, and amplicon sequencing provide a measure of the actual biomass of CB in a sample, as opposed to electrical/chemical activity. This makes them useful, particularly in confirming density at the end of an experiment by comparing to non-destructive methods used throughout an experimental period. However, as EP profiling will only detect the active CB, comparisons to cell quantification methods must be taken with caution as there can be confounding factors in differentiating growing, active, living, and dead CB.

## Geochemical impact

CB have unique geochemical effects on sediments due to their highly specialized metabolism and conductivity, which led to their discovery (Nielsen et al. 2010, Pfeffer et al. 2012, Risgaard-Petersen et al. 2012). Many of the techniques used to measure the effect of CB on their environment are adopted from existing methods, and simply applied in the context of CB research with minimal adaptations (Table 1). These techniques include microprofiling (de Beer 2011, Pfeffer et al. 2012, Marzocchi et al. 2014, Schauer et al. 2014), core slicing to measure profiles (nutrients, dissolved inorganic carbon, iron, etc.) (Sulu-Gambari et al. 2016, Kessler et al. 2018, Hermans et al. 2019, 2020), and analysis of gas from the headspace of closed cores (Scholz et al. 2020).

In the area of geochemistry, the application of these techniques to determine the effects of CB on the environment is what provides new insight. For example, in a comprehensive study on the biogeochemical impact of CB in coastal sediments, both porewater and bottom water were measured for a range of analytes, including cations (inductively coupled plasma—optical emission spectroscopy), dissolved inorganic carbon, iron speciation, and various nutrients (van de Velde et al. 2016). Future studies additionally utilized mass spectroscopy to measure arsenic and other trace metals (van de Velde et al. 2017, 2022), demonstrating the range of biogeochemical analyses that can be done with suitable sample preparation. In cases where the concentration of analyte is very low, such as with trace metals, changing the sampling approach may be required unless highly sensitive equipment is available (van de Velde et al. 2017).

Notably, CB activity affects the pH dynamics of sediment (the ‘Activity’ section), which creates a cascade of effects on the distribution of iron, carbonates, phosphate, and other minerals that have speciation determined in part by pH (Nielsen et al. 2010, Risgaard-Petersen et al. 2012, Meysman et al. 2015). This makes the biogeochemical assessment of CB an impactful area of study, as it helps us fully understand their impact and potential applications in environmental systems.

Another approach from biogeochemistry is isotope tracing, which is often used to answer questions regarding the origin, pathways, and ultimate fates of certain elements within complex ecosystems (Philp 2006). In the study of CB, this allows researchers to disentangle nutrient cycles and determine how CB interact with these elements. Isotope tracing in CB research has focused largely on carbon and nitrogen, and their respective environmental cycles. In particular, ^15^N has been used to study the effects of CB on dissimilatory nitrate reduction to ammonium (DNRA; Kessler et al. 2018), and ^33^P has been used to investigate polyphosphate formations within CB. Although, in the case of phosphorous, the ^33^P isotope is short-lived, so ^18^O-labelled water (H_2_  ^18^O) was measured in conjunction using stable isotope probing (SIP; Geerlings et al. 2022). From this, the formation of polyphosphate granules was hypothesized to assist CB in surviving anoxic conditions, shedding light on the effect of changing geochemistry on CB rather than the other way around.

## Filament extraction

Most of the methods discussed thus far have been centred on macroscale measurements; however, the unique conductivity of CB makes the microscale and nanoscale structures of individual bacteria an evolving area of research. In the attempt to understand how they function and conduct electricity with such high efficiency, the methodologies pivot away from environmental chemistry and draw more upon microscopy, nanoscience, and physics, which typically require isolated filaments. Thus, protocols for the collection of several or single CB filaments have been developed.

### Picking native filaments

To perform these detailed analyses on surface and internal structures, or high-resolution chemical identification, methods have been developed to extract CB from sediment. The main way to achieve this is to pick native CB filaments using hooks. Most often, these hooks are manually made from glass by holding a glass pipette horizontally over a flame until it softens, and then pulling the two ends apart (Li et al. 2022). The temperature of the flame and speed of pulling can be optimized to make glass hooks of different dimensions and shapes. There is not necessarily a ‘perfect’ shape for hooking CB, and it often comes down to the personal preference of the investigator for what type of hook is considered optimal. An alternative to glass hooks is metallic ones, which can be made easily by bending the tip of a syringe needle (Pfeffer et al. 2012). These are less fragile, but can accumulate sediment in the opening of the needle tip, so they may not be as effective for collecting clean CB.

**Figure 7.**
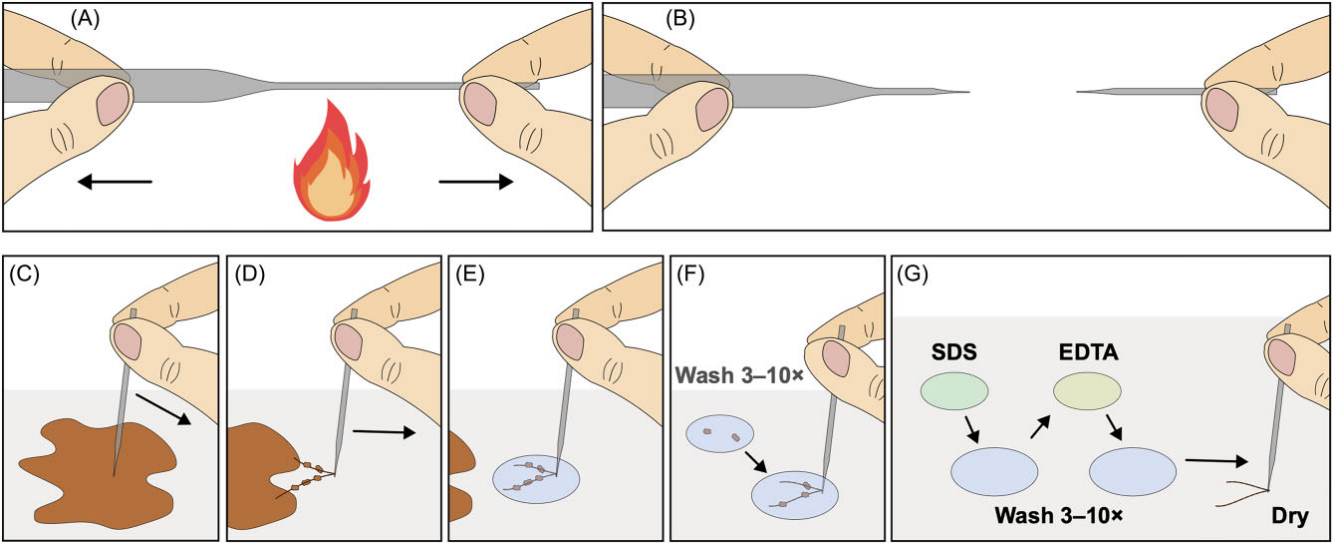
Schematic of the steps involved in extracting and preparing CB filaments using glass hooks to move CB between drops of water and treatment solutions.

The extraction protocol (Fig. 7) is done under a compound microscope, with CB usually appearing as thin fibres being pulled by the hook (Cornelissen et al. 2018, Li et al. 2022). Picked filaments are then thoroughly washed to remove sediment using ultra-pure water. CB can maintain their structure when exposed to osmotic effects due to their resilient cell envelope (Cornelissen et al. 2018). Furthermore, if extracted filaments are to be used for inoculating cores (Fig. 4), they must be pulled through (sea)water (depending on salinity) to minimize exposure to oxygen.

In some cases, particularly with smaller CB (diameter <1 µm), it can be difficult to see the filaments in drops of water, so instead they can be pulled away from the sediment and laid straight onto the glass slide. This ideally leaves a straight, sediment-free section of CB, but due to the lack of washing, there can be more dirt stuck to, or encrusting the cells (Geerlings et al. 2019). Using the droplet rinsing method reduces the presence of sediment and minerals, but is more time-consuming. One consideration in picking CB filaments this way is the bias towards larger species such as *Electrothrix gigas* (Geelhoed et al. 2023), due to the relative ease in extracting these compared to thinner species. As a result, current morphological, genomic, and electrochemical data have been collected from larger CB species (diameter >2 cm).

If the presence of sediment is not a major concern, bundles of filaments can be acquired by gently washing away excess sediment (Risgaard-Petersen et al. 2015). These bundles can be spread out on a microscope slide to allow for observations with light microscopy. Alternatively, centrifugation washing can be done to prepare bulk samples (Geerlings et al. 2019). This involves diluting sediment with ultra-pure water, centrifuging, removing excess water, and repeating (Geerlings et al. 2019). The main aim of this method is to remove salts that could crystalize when the sediment sample is dried down; however, it does not remove the sediment around CB so may not be applicable in many cases.

### Extracting fibre skeletons

An advantage of pulling the bacteria through droplets is that it allows for more specialized treatments. To study the conductive structures of CB (extracted fibre skeletons), a protocol washing CB with sodium dodecyl sulfate (SDS) was developed (Fig. 7). SDS treatment selectively removes lipid membranes and cell cytoplasm, leaving behind the conductive fibres and the periplasmic fibre skeleton in which they are embedded (Meysman et al. 2019). Extracted fibres can further be treated with ethylenediaminetetraacetic acid (EDTA), which acts to complex metals such as calcium and zinc. Removing calcium helps break down cellular protein structures and reduce cell adhesion (Lai et al. 2022), whilst zinc is present in some proteases (Bernkop‐Schnürch and Scerbe‐Saiko 1998a,b), so its removal may prevent the degradation of periplasmic fibres following extraction (Bernkop-Schnurch and Scerbe-Saiko 1998a,b). However, the concentration and incubation time for the EDTA treatment must be carefully monitored (10 min in 1 mM EDTA). EDTA is a chelating molecule, so it can extract metals from a sample, and because the conductive fibres within CB contain metals such as nickel, it is important to minimize the impact of the treatment on the chemistry of the sample itself (Boschker et al. 2021).

As an alternative to picking filaments, there is the potential to combine centrifugation with other treatments such as SDS washing, but it has not yet been shown if washing CB in bulk sediment this way is also effective in removing the cellular membranes. The technique of picking out CB filaments and washing them in drops of various solutions can take some practice to become consistent, and remains time-consuming.

These various extraction techniques have been effective in acquiring samples suitable for a range of analyses, which will be discussed in the following section. However, the complexity in preparing CB highlights the challenge of not being able to culture CB outside of sediment. If CB could be grown in a ‘cleaner’ and more inert medium, the need for extraction would be reduced, and chemical analyses could be performed on bulk samples of bacteria, rather than being limited to low biomass yield from single or multiple filaments.

## Morphology

Following sample preparation, a range of techniques can be used to characterize the surface topology, size, internal structures, and chemical composition of CB. The sample preparation allows for both native filaments and extracted fibre skeletons to be imaged, providing a good range of possible treatments for future experiments. Both established and emerging techniques will be discussed here, and as the study of CB is a rapidly developing area of research, new methods will soon be applied.

**Figure 8.**
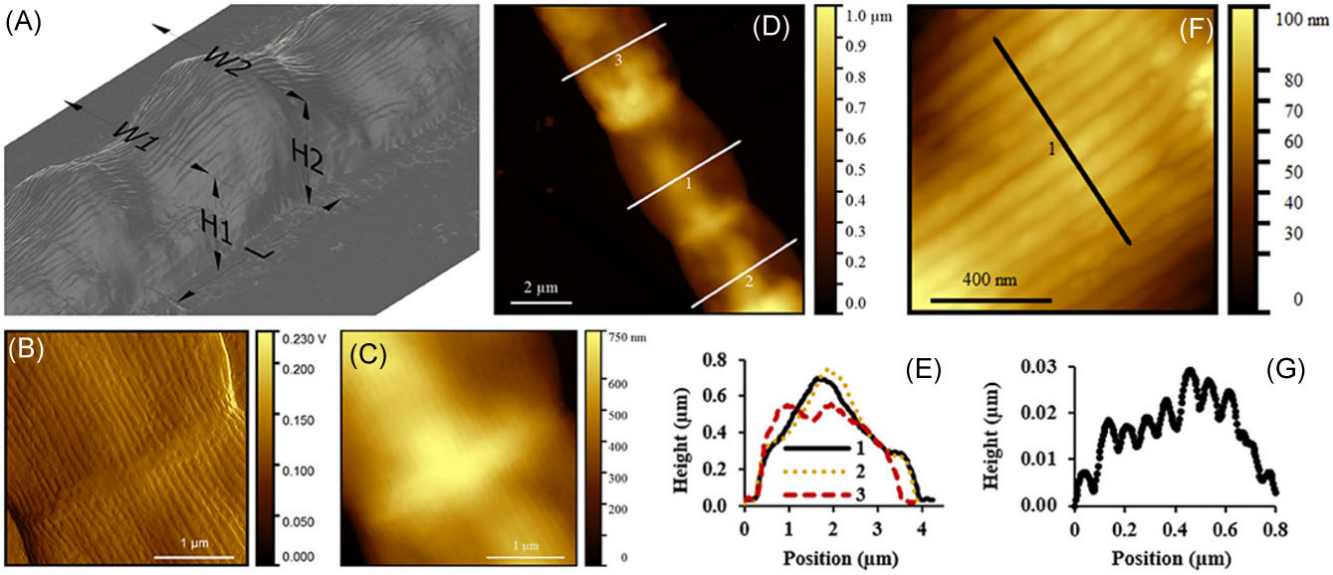
Examples of the types of images that can be acquired using atomic force microscopy on CB. (A) 3D representation of the surface of a filament, (B) error signal image, and (C) the corresponding height measurement. (D) Topography of a larger part of the filament showing cell junctions with (E) cross-section measurements. (F) Image of a smaller area, showing greater detail, and (G) the ridges on the surface of the cell (Cornelissen et al. 2018). Reproduced with permission from Frontiers Publishing under CC BY 4.0 licensing.

Through the application of AFM and electron microscopy (EM), CB have been found to have unique morphological features, with a multicellular structure and ridge pattern (Fig. 8). This allows them to be distinguished from other groups of bacteria. It has also been shown that within CB, different species can have different morphology characteristics, in particular varying cell diameters. For instance, the recently described *Electrothrix gigas* is a particularly large CB species with diameters in the range of 2.5–8 μm (Geelhoed et al. 2023, Sereika et al. 2023). The longitudinal ridges present on all currently known CB species are the most distinguishing feature, as ridge number is highly consistent within a species (Thorup et al. 2021) but varies broadly across CB species.

As several different species of CB have been found in the same sediment (Cornelissen et al. 2018, Marzocchi et al. 2018, Geelhoed et al. 2023), the observation of these morphological variations may become useful in differentiating some of the species.

### Atomic force microscopy

Atomic force microscopy (AFM) is a physical (contact probe) microscopy technique that allows for the topography of a sample to be reconstructed at the nanoscale. Initially, it was used to characterize the surface structure of CB (Marzocchi et al. 2014), showing junctions between cells (Jiang et al. 2018) and the ridges where the conductive fibres run lengthwise in the periplasmic space (Pfeffer et al. 2012). This kind of physical characterization is a standard measurement mode for AFM, and can provide high-resolution images with nanometre scale resolution over areas on the micron scale.

When taking samples of filaments from sediment, AFM can be used to confirm if they are indeed CB or another filamentous organism such as algae. The observation of structures such as cell junctions and the characteristic longitudinal and continuous ridges are considered to be unique to CB (Jiang et al. 2018, Dong et al. 2024), and so can be used as one method of identification. In addition, the diameter of CB can be measured with good accuracy using AFM, and it has been shown that certain species of CB have larger widths than others (Geelhoed et al. 2023, Sereika et al. 2023). Therefore, future experiments may be able to utilize AFM in making estimates of which particular species of CB is present. In analysing AFM images of CB, it is important to note the different image types (depicting different physical information obtained during scanning) that can be produced. Height measurements are the most commonly used, simply showing the relative height from the background (such as a glass slide) to the bacteria (Fig. 8C, D), but other channels, such as error signal, can also be useful. This type of image results from the AFM imaging feedback loop, where rapid changes in height are seen as ‘errors’ corrected by the piezo feedback that controls cantilever motion. Error signal thereby often highlights edge structures more clearly, so it can show the ridges on CB more obviously and allow for easier counting (Fig. 8B). An AFM phase channel (gathered when the AFM is used in alternating contact mode) shows how energy supplied by an oscillating cantilever is dissipated by the surface, indicating mechanical properties (typically hardness versus compliance), so it could be used to determine if a cell is encrusted with clay or iron oxides. The multiple channels of data gained from one image demonstrate the versatility of AFM in observing the surface of CB cells.

In addition to topological characterization, AFM has been utilized to investigate the inner structures of CB. This is a less common use of the technique, and a range of methods have been developed to allow for the imaging of internal structures. The most straightforward is to image extracted fibres (i.e. washed with SDS). By removing the cell membranes and cytoplasm, the conductive fibre skeletons can be directly imaged in high resolution (Cornelissen et al. 2018). Another, more novel application of the technique is using the AFM itself to do *in vitro* dissection. This involves scraping away the surface of the cell with the AFM cantilever, exposing inner structures or cutting specific parts of the cell. This was used to great effect in identifying the conductive structures within CB (Jiang et al. 2018). Furthermore, by damaging one half of the cell, and utilizing conductive AFM (C-AFM) to measure the local conductivity on the sample, researchers were able to trace the pathways of conductivity in the CB and link them to the previously observed fibres embedded in the ridges (Thiruvallur Eachambadi et al. 2020). Whilst similar dissections could be performed by laser ablation, AFM tends to be more precise and less damaging to structures of interest (Stark et al. 2003).

This also highlights that, for a species of bacteria with highly conductive fibres, C-AFM is a valuable technique. Early in the study of CB, failure to acquire evidence of conductivity through C-AFM challenged the hypothesis of electron transport through the fibres over long distances (Nielsen and Risgaard-Petersen 2015, Meysman 2018). However, in the time since those initial investigations, it has been found that the conductivity within CB is highly sensitive to oxygen, so any conductivity experiments (including C-AFM) must be done in an oxygen-free atmosphere. By optimizing these measurement conditions, C-AFM can show conductive pathways, and how they can be affected within CB by cutting paths using the AFM cantilever (Thiruvallur Eachambadi et al. 2020), which is of interest as the field moves towards deeper understanding of the mechanism of conductivity.

The AFM techniques highlighted so far are performed with cells on surfaces in ambient air or inert atmosphere in the case of C-AFM. This may result in a slightly distorted image of the structure of CB as they typically live in an aqueous environment, and so likely dehydrate when taken out of that context. In-liquid AFM imaging can be performed to provide information on CBs’ more natural structure. Using in-water AFM also allows for adhesion force measurements, providing insight into the interactions between CB and surrounding particles. However, this has not yet been reported, showing that the use of AFM in CB research has a range of applications yet to be explored. Furthermore, combining AFM with other techniques is proving an innovative way to tackle specific challenges in this field. For example, a recent study used co-located time-of-flight secondary ion mass spectrometry and AFM to identify the layered structure of the periplasmic fibres and their carbohydrate sheath using a specially designed setup (Thiruvallur Eachambadi et al. 2021).

### Electron microscopy

In CB research, electron microscopy is often used as a complementary technique to AFM. Scanning electron microscopy (SEM) provides similar information on the topological structures of samples to AFM by measuring electron density and using the reflected electrons to build up an image. One of the major advantages of SEM is that it has better lateral resolution than AFM in most setups. Standard SEM can resolve structures down to nanometre scale, whilst AFM is limited by the size of the cantilever used (often around 30 nm). Whilst AFM in theory can achieve atomic resolution, this is extremely challenging to achieve and requires very clean samples, which CB are typically not.

This difference in lateral resolution is one of the factors that makes SEM an attractive technique in some cases. Even when looking at larger features of CB, SEM has still proven to be useful. For example, it was used to first observe bundles of CB and their interaction with solid electrodes (Reimers et al. 2017). This would have been more challenging on AFM due to the 3D nature of the sample. Furthermore, focused ion beam SEM and TEM allow for cross sections of individual filaments to be imaged, typically by fixing and embedding cells in resin followed by microtoming. These methods provide functionality that AFM cannot easily provide.

In addition to topological imaging, SEM has different modes that allow for a wider range of analyses. Energy dispersive X-ray (EDX) analysis is commonly performed to identify the presence and relative abundance of certain elements, particularly heavier ones such as metals. This has been used previously to identify the presence of iron oxides encrusting CB filaments (Geerlings et al. 2019), and to crudely detect other metals within the conductive fibres such as nickel (Digel et al. 2023).

Aside from SEM, electron microscopy offers a wide range of other methods for observing structures within CB. A complementary technique is transmission electron microscopy (TEM), which, instead of using reflected electrons to construct an image, uses the electrons that have passed through the sample. As a result, TEM is better for observing internal structures as opposed to topological features. To get clear TEM images, the sample being imaged must have a thickness small enough to allow sufficient electrons to reach the detector. Depending on the CB being used, they may need to be cut (with a microtome or similar method) to create slices thin enough for this technique, usually around 200 nm, but some CB have been imaged intact with excellent results (Trojan et al. 2016). TEM can also be used with cryo-electron tomography to build up 3D models of internal structures such as vesicles, membranes, and more recently, chemosensory arrays (Cornelissen et al. 2018, Digel et al. 2023). TEM highlights areas of high contrast, so it may be particularly useful in identifying the locations where metals exist within CB, potentially elucidating some of the structures making up the conductive fibres.

Within electron microscopy there is a wide range of more specialized techniques, each of which may provide unique insight into the composition and structure of CB. The use of cryogenics is one of the more common and accomplishable extensions of both SEM and TEM, and allows for the bacteria to be flash frozen in their native state (hydrated), rather than the usual dehydrated form required at the low pressures of EM (Digel et al. 2023). Environmental SEM takes this idea further by keeping samples at a relatively high pressure through the presence of water vapour (Donald 2003). In the case of CB, this would allow cells to stay hydrated without the need for cryogenics, and thus maintain more of their native structure for analysis.

### Comparison of AFM and EM for CB study

For the more common uses of AFM and EM in CB research, the two modes of analysis often provide very similar results. The obvious exceptions to this are force measurements with AFM and most uses of TEM in observing internal structures, but in studies that are not using either of these specialized applications, it is worth considering the pros and cons of AFM and EM. The main factors to consider are sample preparation, area of interest (surface structure or composition), and equipment accessibility.

The size scale of CB is fairly large in the scheme of nano-characterization techniques, so whilst SEM can provide higher resolution more easily compared to AFM, this may not be necessary depending on the structures of interest. In particular, when looking to identify CB in a sample, or measure diameter, both methods are adequate and differ only in ease of access and sample preparation. SEM tends to be more widely used because it is seen as being applicable in a broader range of studies (across materials science generally, not just in CB research), but the sample preparation can be more involved. Whilst AFM requires very clean samples to get good images, EM samples must be coated with a conductive layer of metal before imaging, requiring a sputter coater. In addition, due to the intense electron beam, SEM is destructive to most samples, whereas AFM is not. Therefore, for repeated analysis of the topography of a CB filament, AFM would be the preferred method as it is less perturbative.

When choosing how to observe the surface of CB for an experiment, these are just some of the factors that should be considered. In many cases, the two methods can be used to complement each other and build a more robust data set, but EM should always be performed second due to its more destructive nature.

## Electrochemical and electrical analyses

### Electrochemical analysis

Due to the highly electroactive nature of CB, electrochemical analysis has been an area of particular interest. However, preparing and analysing samples for these experiments have required extensive problem-solving over the years due to the challenges of acquiring bulk samples. Now that cleaning CB (native filaments) and extracting the conductive fibre skeleton is a well-established methodology, the oxygen sensitivity is well known (although not yet well understood), and measuring the properties of these microbes has become more straightforward.

Most of the electrochemical studies thus far have involved depositing bundles of native filaments, and in some cases extracted fibre skeletons, onto gold electrodes to use as the working electrode in cyclic voltammetry (Meysman et al. 2019, Geerlings et al. 2020), or placing electrodes into sediment with and without active CB (Reimers et al. 2017, Bonne et al. 2024). The first method facilitates measurements of the electrochemical properties of clean, intact CB, whereas the second measures the properties of the sediment and how CB affect that environment (Algar et al. 2020). One advantage of performing electrochemical measurements on extracted CB fibre skeletons is that it allows for detailed characterization of the redox properties of the conductive fibres. However, at present there has been little progress in understanding any potential electrochemical factors in the long-distance or extracellular conductivity of CB, aside from the measurements of degradation when exposed to high oxygen concentrations (Meysman et al. 2019) and the catalysis of oxygen reduction at low concentrations (Geerlings et al. 2020).

### Conductivity

Electrical measurements are often coupled with electrochemical techniques, but rather than providing insight into the contribution of chemical changes through redox processes, they measure conductivity directly. This has been done through placing native filaments onto non-conductive surfaces patterned with conductive regions (Meysman et al. 2019). Here, the CB filament acts as a bridge between two conductive regions, enabling the current to be measured across the bacteria. When performing this type of experiment, it is important to make sure the bacterial filament is well connected to the two conductive regions. This also applies to techniques such as C-AFM, and to ensure good connection, carbon paste can be applied to both ends to make good contact with the conductive surface (Thiruvallur Eachambadi et al. 2020).

Similarly to electrochemical techniques, these can be done on both native CB filaments and extracted fibre skeletons, with the properties of the two being somewhat different. For example, the conductivity of extracted skeletons was found to be more resistant to oxygen degradation than native filaments, raising questions about the mechanism of conductivity within CB and to their surroundings (Meysman et al. 2019, Pankratov et al. 2024). However, a thorough understanding of these differences has been somewhat challenging. More recent studies have utilized EM techniques to observe the conductive fibres in higher resolution, in combination with direct conductivity measurements, to work towards a deeper understanding of this aspect of CB (Digel et al. 2023).

There has also been recent work in understanding the physics driving the conductive mechanisms of CB. This has largely been done through modelling rather than experimental approaches, and was able to provide insight into the quantum mechanics of this novel bioconductivity (van der Veen et al. 2024). Combining models with observations of a temperature dependence (Bonne et al. 2020), an electron hopping mechanism has been proposed. The corroboration of each result highlights the value of multidisciplinary approaches in CB research.

### Chemical composition

There are a wide range of nanoscale chemical techniques that can be applied to understand the chemical composition of extracted native CB and fibre skeletons. As most do not require adaptations specific to CB, an example is presented here where multiple forms of analysis were utilized to gain complementary information (Boschker et al. 2021). By combining results from Raman microscopy, AFM-based infrared spectroscopy, scanning dielectric microscopy, synchrotron X-ray microscopy (XRM), X-ray fluorescence (XRF), and mass spectrometry, a more complete understanding of the composition and structure of the novel conductive fibres within CB was achieved.

In this study, Raman spectroscopy and AFM-IR were both applied to gain complementary information. Raman microscopy had previously been used to identify resonance bands of cytochromes on CB (Bjerg et al. 2018), and here the same bands were observed again, with a few additional ones, indicating the types of chemical bonds that may be present. AFM-IR was then able to provide further insight into the types of bonds and their location.

The application of synchrotron radiation (XRM and XRF) is also an innovation in CB research, showing that the high-intensity beams emitted from a synchrotron are highly applicable to microbiological samples (Boschker et al. 2021). Synchrotron radiation is most beneficial for samples that are too small or contain very low concentrations of analytes (e.g. metal atoms within CB) that cannot be measured on benchtop X-ray equipment. Due to methodologies currently being limited to measuring individual CB filaments, this allowed for the observation of metals such as nickel and copper within the conductive fibres and membranes, which cannot be easily measured with conventional methods.

Many other analytical chemistry techniques may be useful in measuring the types and locations of element or chemical bonds within CB. However, one study (Bjerg et al. 2018) highlighted some crucial analytical techniques, which, e.g. have led to the discovery of nickel cofactors within CB fibres, furthering our understanding of the structure and composition of this novel material.

## Physiology

### Metabolism

Whilst there is a well-developed understanding of the metabolism of CB on a macroscopic level (sulfide oxidation and oxygen/nitrate reduction), studying their metabolism on a cellular level remains challenging due to the lack of axenic cultures in artificial media. Despite this, methods that look at the cellular level, such as genomics and nanoscale secondary ion mass spectrometry (nanoSIMS), have had success in improving our understanding of CB metabolism, whilst other techniques such as enzyme assays and metabolomics have not yet been applied. Further research is required to unravel the metabolism of CB and identify the functions of hitherto unknown genes, many of which may be unique to the CB lineage.

#### Genomics

Upon collecting a sample from the environment, enrichment cultures, or picked CB filament, sediment and filaments are stored at −80°C, prior to DNA extraction and genome assembly. It has been suggested that the rigid outer structure of their cell envelope may impair the extraction of DNA at the cell lysis step, making it less efficient for CB in comparison to other bacteria (Pfeffer et al. 2012, Trojan et al. 2016, Reimers et al. 2017). However, there has been success with commercial DNA extraction kits as well as enzymatic DNA extraction when applied to CB within sediment (Trojan et al. 2016, Geelhoed et al. 2020, Sereika et al. 2023, Hiralal et al. 2024a,b, Plum-Jensen et al. 2024), suggesting that the rigid cell envelope does not hinder these methodologies. In cases where low amounts of DNA material are targeted in an extraction, commercial whole genome amplification kits are recommended (Hellani et al. 2004). For CB, this has allowed for closed genomes to be generated from single filaments (Kjeldsen et al. 2019, Geelhoed et al. 2023, Hiralal et al. 2024a,b).

Following extraction, the sequencing and analysis of DNA requires few modifications for CB compared to other bacteria, using established sequencing platforms such as Illumina, PacBio, or Nanopore. Sequences are assembled into draft genomes if extracting DNA from a single filament (Kjeldsen et al. 2019), or into metagenome-assembled genomes from bulk sediment samples using a combination of short-read and long-read sequences (Sereika et al. 2023, Hiralal et al. 2024a,b).

When analysing genomes, comparative genomics methods can provide insights into gene functions and metabolic potential of microbes (Hiralal et al. 2024a,b, Plum-Jensen et al. 2024). CB have a high number of unknown genes (>50%) in their genomes, for which exact functions remain to be identified (Kjeldsen et al. 2019), making comparative analyses challenging. However, an example of where this approach has provided insights on CB is the discovery that the genomes of all *Electrothrix* species and *Electronema halotolerans* contained one or more copies of the H^+^/Na^+^ antiporter gene *nhaA*, suggesting that these antiporters may be an adaptation to brackish and marine environments and facilitate osmoregulation (Kjeldsen et al. 2019, Dam et al. 2021, Sereika et al. 2023).

Some studies have applied other omics techniques such as transcriptomics and proteomics (Kjeldsen et al. 2019, Marzocchi et al. 2022). Both these omics techniques hold strong potential to contribute to our understanding of CB metabolism. Metatranscriptomics, metaproteomics, and metabolomics, in particular, are applicable due to their capacity to analyse multi-species samples (Jansson and Hofmockel 2018). With CB cultures often containing multiple strains, these techniques can provide insight into common features within the diversity of CB, as opposed to focusing on individual filaments. Furthermore, as discussed in Zhang et al. (2019), each approach provides a different level of information, such as determining not just genetics, but which genes are being expressed.

Using metatranscriptomics, it was shown that freshwater CB were capable of dissimilatory nitrate reduction to ammonium (DNRA; Marzocchi et al. 2022). Proteomic data allowed for the detection of several key enzymes, and may lead to the identification of proteins involved in the electrical conduction mechanism in the future (Kjeldsen et al. 2019).

However, challenges present in these approaches include the microbial and chemical complexity of sediment. The quality of DNA, RNA, or proteins obtained from sediment can limit the level of detail and accuracy of results. As a result, combinations of molecular sequencing and microbial community measurements (Jessup et al. 2005) may continue to be utilized to provide in-depth understandings of CB, their metabolism, and their place in the ecosystem.

#### Stable isotope labelling and NanoSIMS

One method applied to study CB growth, cell division, and reproduction is nanoSIMS. In one case, ^13^C-labelled biomolecules were doped into CB enrichment cultures, but rather than measuring their reaction pathways from sediment analysis (the ‘Geochemical impact’ section), nanoSIMS allowed to track the incorporation of the ^13^C isotopes within individual CB (Vasquez-Cardenas et al. 2015).

NanoSIMS, biorthogonal noncanonical amino acid tagging, and isotope labelling further revealed a division of labour between cells in CB filaments (Kjeldsen et al. 2019, Geerlings et al. 2020, 2021). Only cells located in the anoxic sediment layer are capable of biosynthesis and energy conservation, whilst cells in the oxic layer reduce electron acceptors without contributing to biosynthesis or growth of the filament. This was shown by measuring the assimilation rates of ^13^C-bicarbonate and propionate into the cells, indicating that CB are facultative chemoautotrophs (Geerlings et al. 2020). A more recent study using nanoSIMS studied the growth of CB using dual-label SIP (^13^C and ^15^N) nanoSIMS, showing that cell growth and division are strictly controlled by LDET and the access to oxygen (Geerlings et al. 2021), showing that this method is still highly relevant in the field.

In contrast to nanoSIMS being used to study cell growth on an individual scale, measuring CB growth rates in an overall incubation can effectively be done using FISH as discussed previously. For example, doubling times of 20 h for CB have been determined using FISH counts and carbon isotopes, allowing for comparisons between CB and the doubling time of other microorganisms (Schauer et al. 2014, Vasquez-Cardenas et al. 2015). However, this approach does not measure the growth of individual filaments or cells.

Further study of the growth of CB and their metabolism on both cellular and whole-filament levels may include these main methods; however, a range of other techniques can also be applied depending on the approach. Planar optodes can be used to measure analytes such as O_2_ in sediments and microbial mats (Glud et al. 1996), and have been successfully utilized in CB research to find that generally only 10% of the cells within a filament are connected to oxygen, whilst 90% of the cells grow and conserve energy (Scilipoti et al. 2021). AFM data have also been used in other studies to propose a model for the growth and cell division of CB (Cornelissen et al. 2018, Jiang et al. 2018).

If and how CB exist in a single-cell stage remains unclear. Few species-specific growth or cell division rates have been reported with the notable exception of depth-resolved estimates from amplicon sequence variants (Liau et al. 2022), but with the increasing numbers of single-species cultures available, there may be more development in this area. A new study has suggested a minimum filament length of 15 µm (∼5 cells) for CB to colonize a new section of sediment (van Dijk et al. 2024), showing progress being made in understanding the growth of CB.

### Motility

Due to the opacity of sediment, CB cannot be directly observed in their natural environment, so sandwich and ‘trench slides’ were developed to allow for more direct observation of their movement and behaviour. Motility had previously been determined through changes in the geochemical fingerprint as oxygen supply was altered (Malkin and Meysman 2015), but the ability to directly observe live CB has proven extremely valuable.

Trench slides create a limited pathway for CB to access oxygen, forcing them out of the sediment, allowing them to be observed with optical microscopy (most commonly phase contrast). There are a range of trench slide designs with slightly different applications, described in an initial study by Bjerg et al. (2016). The simplest is created by placing sediment on a glass slide and ‘sandwiching’ it with another slide. The space between the two slides can then be flushed with water (using appropriate salinity), which both removes any loose particles and provides the medium for CB to move through. The layer between the two glass slides will vary in thickness depending on the amount of pressure applied, but is typically in the hundreds of microns scale, limiting the area for oxygen to diffuse into from the edges significantly.

**Figure 9.**
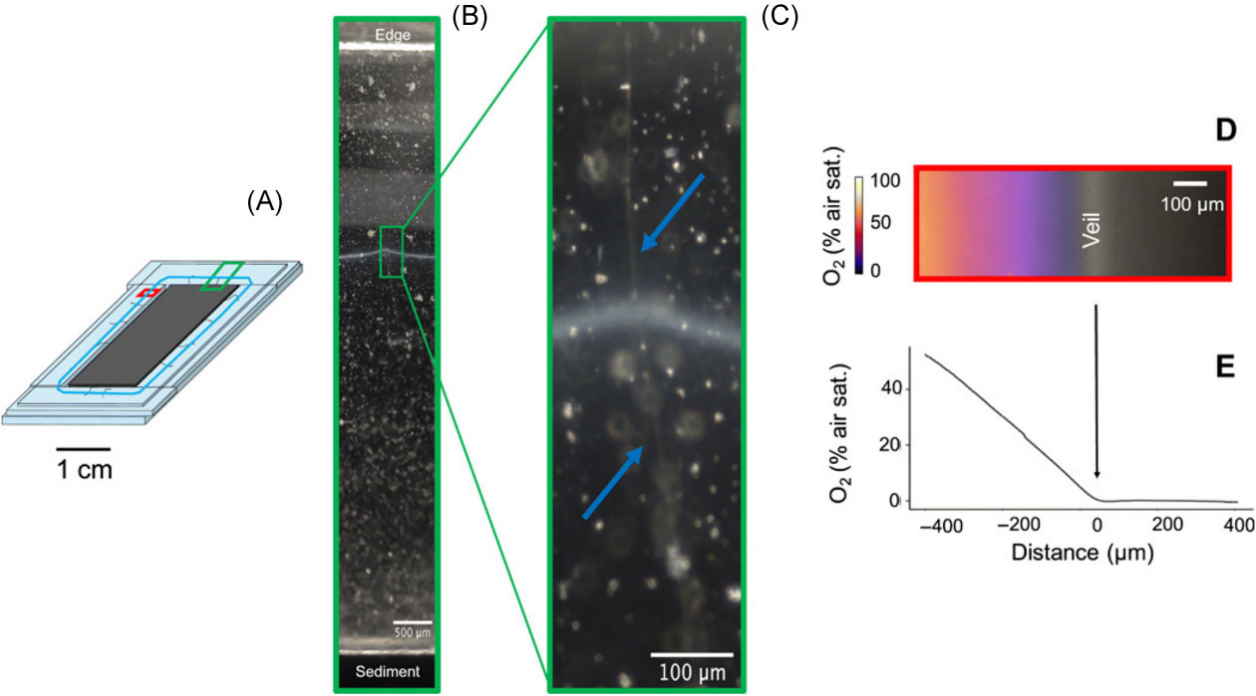
(A) Diagram of a trench slide setup with CB emerging from sediment, and dark-field microscopy images of the clear part of the slide showing the microaerophilic veil (B) with a CB filament crossing through the boundary (C). (D) shows the oxygen gradient using planar optodes and (E) the corresponding oxygen profile (Scilipoti et al. 2021). Reproduced with permissions from Science Journals under CC BY-NC 4.0 licensing.

To make a more robust setup, a trench can be created by gluing together pieces of glass slides as shown in Fig. 9A. This provides a well-defined sediment edge, making identification of CB migrating out of this region easier. The principle behind the trench slides is the same as with the ‘sandwich’ slide; by limiting oxygen influx into the water, CB are forced to migrate out of the sediment trench towards the edges of the glass slide (Bjerg et al. 2018). As glass is optically transparent, the CB can be observed whilst alive and active, which is not possible within sediment. The trench slide has become the most common method used to study individual, living CB, as it is fairly straightforward to set up and allows for the observation of the bacteria on timescales ranges from hours to weeks. The rate of movement will vary greatly depending on the exact setup as the thickness, amount of sediment, and microbial activity will all contribute to the rate of oxygen consumption within a trench slide. In experiments observing CB and their interactions with other microbes, 1 day was allowed for concentration gradients to establish before taking measurements (Bjerg et al. 2023, Lustermans et al. 2023). Although CB might migrate out of sediment within a couple of hours, it can be beneficial to let the system reach equilibrium. This equilibrium can often be directly observed through a microaerophilic veil (Fig. 9B–D), which is a cluster of microorganisms that migrate to the oxic–anoxic interface, creating a useful visual distinction of the border (Lustermans et al. 2021).

In addition to the ‘sandwich’ and more common ‘trench’ slides, a range of variations have been created to mimic different concentration gradients or guide the direction of movement of CB. An example is adding nutrient reservoirs into holes drilled into the glass, which can then be used to explore the drivers of motility for CB (Bjerg et al. 2016).

Whilst trench slides were devised to investigate the mechanism behind CB motility, these innovations have made them useful in much wider applications, including the exploration of CB interactions with their environment, solid surfaces, other microbes, and oxygen consumption (Scilipoti et al. 2021, Bjerg et al. 2023, Lustermans et al. 2023). The use of trench slides has enabled the discovery of a multitude of bacteria that create flocks around CB filaments in anoxic sediment, and utilizing lasers to cut the filaments showed the dispersion of these flocking bacteria indicating possible electron transfers between CB and other species (Bjerg et al. 2023). Raman microscopy of living CB in trench slides was also applied, where a gradient in cytochrome redox potential was discovered that was dependent on the oxygen access of a filament, providing direct evidence for LDET (Bjerg et al. 2018). This highlights the versatility of trench slides, which then allows for a much deeper understanding of the chemotactic response of CB, and expands the potential for future research into their behaviour and requirements to survive. The inconsistency in construction dimensions and sediment volume are relatively minor drawbacks of trench slides in quantifying motile CB, and they remain highly effective and widely utilized in the field.

#### Potential alternatives

There has been minimal exploration in the literature for alternative methods of studying CB motility, but this does not mean that trench slides are the sole method. One example of potential alternatives is the use of TTC agar, a commonly used technique in diagnostic microbiology (Ball and Sellers 1966). This is an agar medium made with 2,3,5-triphenyltetrazolum chloride, which is colourless but can be reduced to formazan through bacterial metabolic pathways, concomitantly turning a red colour. In typical applications, the agar is made in tubes, and stab lines of a bacterial sample are inserted, and the agar colour acts as an indicator for whether or not the bacteria are motile (Ball and Sellers 1966). This method may be challenging for CB due to the presence of other microbes in sediment, but it could be modified to indicate the movement of CB towards or away from added reagents in regions of the agar tube. This could also allow for greater control over the concentration gradients. Although trench slides are limited to oxygen-sulfide, an agar tube could have other compounds added and be kept anoxic. In addition, the viscosity of agar can be adjusted to mimic the porosity of sediment to help maintain desired concentration gradients whilst allowing for the movement of bacteria throughout the medium. There is also precedent for CB living in agar, provided the sulfide concentration gradient is maintained (Sachs et al. 2022), opening up the possibility to grow CB in artificial media (Stiefelmaier et al. 2024).

Studying the motility of CB may become more important as the field moves towards understanding and applying the unique conductivity of these organisms. Trench slides are one highly innovative and adaptable solution, and considering alternative methods of observing the motility and migratory behaviour of CB would allow for more robust experimental designs in the future.

## Diversity

### Identification and taxonomy

Given that morphological differences are not sufficient to distinguish between species of CB (the ‘Morphology’ section), the use of molecular techniques is warranted. Due to advances in molecular biology, bacterial taxa can be identified precisely down to the species and strain level based on their genetic variation. This level of detail has allowed for several CB species to be validated under the ‘Code of Nomenclature of Prokaryotes Described from Sequence Data’ (SeqCode), removing the ‘*Candidatus*’ designation of species for which high-quality genomes are available (Hedlund et al. 2022, Plum-Jensen et al. 2024).

DNA or RNA sequencing are techniques required to identify CB taxonomically and investigate their phylogeny, and all currently described and potential new CB species were classified taxonomically by either their 16S rRNA genes or their whole genomes (Trojan et al. 2016, Kjeldsen et al. 2019, Thorup et al. 2021, Geelhoed et al. 2023, Sereika et al. 2023). The known diversity of CB has grown significantly since the first taxonomic framework was presented, and still likely remains underestimated.

#### 16S rRNA gene

Described and potential new CB species can be identified by sequencing the 16S rRNA gene of individual CB filaments (partial or full-length 16S rRNA gene) or by targeting all 16S rRNA genes present in a bulk sample. Commonly used threshold values for species and genus delineation in bacteria are 98.7% and 94.5% nucleotide identity of the 16S rRNA gene, respectively (Yarza et al. 2014).

Amplicon sequencing of the 16S rRNA gene has been widely used to characterize microbial communities in CB enrichments and field samples (Scholz et al. 2020, Dam et al. 2021, Liau et al. 2022, Hiralal et al. 2024a,b, Plum-Jensen et al. 2024). Hypervariable regions that have been targeted are V3-V4, V4, and V4-V5, resulting in partial 16S rRNA gene sequences typically allowing to identify taxa at the genus level (Geelhoed et al. 2020, Dam et al. 2021, Liau et al. 2022). Community analysis allows for the identification of CB, and potentially obligate associated microbes (Vasquez-Cardenas et al. 2015, Dam et al. 2021, Thorup et al. 2021).

A straightforward method to identify a CB species is by separating a single filament from sediment, PCR-amplifying, and sequencing the (almost) complete 16S rRNA gene (Marzocchi et al. 2014, Schauer et al. 2014, Yang et al. 2024). Full-length 16S amplicon sequencing by long-read sequencing platforms such as Nanopore and PacBio can provide higher taxonomic resolutions (Xu et al. 2021a,b, Sachs et al. 2022) with the PacBio sequencing platform being used to obtain full-length 16S sequences of CB and their surrounding microbial communities (Xu et al. 2021a,b, Sachs et al. 2022).

Once the 16S rRNA gene or other suitable target genes of a CB species are known, species-specific FISH probes can be designed and used to identify and quantify specific CB species within sediment (see the ‘Fluorescence *in situ* hybridization’ section) (Marzocchi et al. 2014, Geelhoed et al. 2020, 2023).

#### Whole genomes

Next to phylogenetic marker genes, whole genome sequencing has become a valuable tool in studying microbial diversity, allowing for a higher taxonomic resolution of taxa compared to 16S analysis (Lang et al. 2013, Hugenholtz et al. 2016). To delineate bacterial species, whole-genome average nucleotide identity is used (cut-off 95%), whilst whole-genome average amino acid identity is applied to delineate genera (cut-off 65%) (Konstantinidis et al. 2017).

If the genome quality is sufficiently high in terms of assembly contiguity, contamination, and the presence of conserved genes, it may be possible to assemble the 16S rRNA gene from genome sequencing data. In the case of CB, this has only successfully been done with single-strain cultures and well-constructed experimental pipelines (Sereika et al. 2023, Hiralal et al. 2024a,b, Plum-Jensen et al. 2024). In general, 16S rRNA gene sequences from metagenomic data are difficult to assemble and are often not binned with the other contigs of a taxon due to distinct nucleotide frequencies (Yuan et al. 2015).

Similarly to marker genes, whole genomes can be sequenced both from single filaments and sediment samples. However, sequencing whole genomes from single CB filaments requires a whole genome amplification step (Trojan et al. 2016, Geelhoed et al. 2023). Genomes from single filaments have historically not been as high quality as genomes from sediment samples, but this may also be due to the constant improvement of sequencing methods over time (Trojan et al. 2016, Sereika et al. 2023).

### Phylogeny

The phylogeny and evolutionary relationships of CB can be investigated by analysing phylogenetic trees, which can be constructed from multiple sequence alignments of nucleotide or protein sequences (Trojan et al. 2016, Kjeldsen et al. 2019, Geelhoed et al. 2023, Sereika et al. 2023, Hiralal et al. 2024a,b, Plum-Jensen et al. 2024).

The highly conserved 16S rRNA gene is the most widely used marker gene to reconstruct prokaryotic phylogenies. Whilst the commonly used V3-V4 region (ca. 440 bp) of the 16S gene is not suitable to assign taxonomies at the species level, it can indicate the phylogenetic placement of a taxon in a phylogenetic tree, albeit with lower resolutions than full-length 16S sequences (≥1400 bp) (Kim et al. 2011, Jeong et al. 2021). Many bacteria, including known CB species, have multiple copies of the 16S gene in their genome. Consequently, intragenomic variation between 16S copies may affect taxonomic resolution and should therefore be considered when determining species and strain levels (Johnson et al. 2019). Another robust marker gene that has been targeted to study CB phylogeny is the *dsr*AB gene (Müller et al. 2015, Trojan et al. 2016).

More recently, phylogenomic trees have taken into account 120 bacterial marker genes, which allowed for a more accurate inference of evolutionary relationships between CB (Kjeldsen et al. 2019, Geelhoed et al. 2023, Sereika et al. 2023). In the future, phylogenetic and phylogenomic analysis may unravel the evolutionary link between the two described CB genera and identify the first lineage that adopted an electrogenic metabolism (Kjeldsen et al. 2019, Sereika et al. 2023).

## Future directions

As the study of CB is relatively new, the methods used to study them are constantly being developed or adapted from existing techniques. As described throughout this review, established techniques used in fields such as biogeochemistry or genomic analysis have typically been transferable to CB research with minimal alterations, whilst other aspects such as electrical and electrochemical analyses have required significant innovations, particularly with sample preparation and manipulation, to advance the field.

There remain areas of CB research that would benefit from further development as highlighted throughout. In particular, the optimization of culturing methods would enhance consistency between studies. The development of single-strain cultures was a major step in this area (Thorup et al. 2021), showing that progress can be made; however, we are still far from an ‘ideal’ CB culture that would allow them to grow in more simplified media, as an axenic or co-culture. The observation of CB in agar pillars within sediment cores indicates a possible direction for the development of CB cultures that are easier to extract and analyse CB from (Sachs et al. 2022) through the extraction and dissolution of agar. Alternatively, utilizing synthetic sediments would reduce variability between experiments for geochemical research (Stiefelmaier et al. 2024) and increase the density of CB, allowing for more efficient extractions. It may be that finding ways to achieve this, such as the addition of sulfide to autoclaved enrichment cultures (Xu et al. 2021a,b), would sufficiently improve our ability to collect bulk, clean CB for chemical or genomic analysis, bypassing the need for axenic cultures.

Currently, the methods used for growing CB enrichments may be a cause for the limited ways to measure their activity and density. Most literature has described the use of microprofiling and FISH to measure activity and density, but this does not mean the accuracy of these measurements cannot be improved. Conversely, this consistency in methods has allowed for comparison between different sediments, as well as common experimental workflows in CB experiments (Fig. 2). If new quantification techniques were to be developed, combining them with these established methods in an experimental design would be beneficial. Additionally, reporting the ratio between different quantification approaches, such as RNA:DNA (Liu et al. 2021, Liau et al. 2022), or correlating current density to CB abundance (van de Velde et al. 2016), can lead to better comparison and increase accuracy in activity and density estimations.

Aside from developing new techniques to improve on existing methodologies, there is also the capacity to leverage emerging and cross-disciplinary technologies to gain deeper understanding of these unique organisms. Trench slides are an example where innovation and development of new methods allowed for the rapid expansion of CB research through direct observations of living filaments. Furthermore, it allowed for techniques such as Raman microscopy, or even the characterization of surrounding microbes, to be applied where it had not previously been possible. The combination of methods has been a growing area that shows much promise and allows for many possibilities in the future as the field becomes increasingly multidisciplinary (Thiruvallur Eachambadi et al. 2021, Bjerg et al. 2023). In gaining new insight into the properties and physiological mechanisms of CB, it may be that utilizing emerging and interdisciplinary methods becomes necessary. In particular, molecular biochemistry methods such as genomics and proteomics rapidly develop in accuracy and ability to work with small biomass (such as single CB filaments), so the application of new protocols may allow for new findings within CB genomes.

Having a broad range of methods available is crucial to performing robust science on such a unique group of bacteria. Both approaches of applying established methods in new ways and developing new methods that exploit the unique characteristics of CB have distinct advantages and allow for better experimental designs in the future, enabling researchers to contribute to a deeper understanding of CB, their impacts, and potentials. The unique conductivity and metabolism of CB make them a highly intriguing target for future research, as their conductive fibres may in the future allow for biological circuits, biodegradable electronics, and a range of other applications (Bonne et al. 2022). This makes ongoing research vital in furthering our understanding of CB, their potential applications, environmental effects, and their uniquely conductive fibres.
